# Inflammatory pathways in COVID‐19: Mechanism and therapeutic interventions

**DOI:** 10.1002/mco2.154

**Published:** 2022-08-01

**Authors:** Yujie Jiang, Tingmei Zhao, Xueyan Zhou, Yu Xiang, Pedro Gutierrez‐Castrellon, Xuelei Ma

**Affiliations:** ^1^ Laboratory of Aging Research and Cancer Drug Target State Key Laboratory of Biotherapy National Clinical Research Center for Geriatrics West China Hospital Sichuan University Chengdu PR China; ^2^ Department of Biotherapy State Key Laboratory of Biotherapy Cancer Center West China Hospital Sichuan University Chengdu PR China; ^3^ Center for Translational Research on Health Science Hospital General Dr. Manuel Gea Gonzalez Ministry of Health Mexico City Mexico

**Keywords:** COVID‐19, cytokine storm, immunopathology, immunotherapy, inflammatory pathways

## Abstract

The 2019 coronavirus disease (COVID‐19) pandemic has become a global crisis. In the immunopathogenesis of COVID‐19, SARS‐CoV‐2 infection induces an excessive inflammatory response in patients, causing an inflammatory cytokine storm in severe cases. Cytokine storm leads to acute respiratory distress syndrome, pulmonary and other multiorgan failure, which is an important cause of COVID‐19 progression and even death. Among them, activation of inflammatory pathways is a major factor in generating cytokine storms and causing dysregulated immune responses, which is closely related to the severity of viral infection. Therefore, elucidation of the inflammatory signaling pathway of SARS‐CoV‐2 is important in providing otential therapeutic targets and treatment strategies against COVID‐19. Here, we discuss the major inflammatory pathways in the pathogenesis of COVID‐19, including induction, function, and downstream signaling, as well as existing and potential interventions targeting these cytokines or related signaling pathways. We believe that a comprehensive understanding of the regulatory pathways of COVID‐19 immune dysregulation and inflammation will help develop better clinical therapy strategies to effectively control inflammatory diseases, such as COVID‐19.

## Graphical abstract

The 2019 coronavirus disease (COVID‐19) pandemic has become a global crisis, with SARS‐CoV‐2 infection inducing excessive inflammation and cytokine storm. Cytokine storm leads to acute respiratory distress syndrome (ARDS) and multi‐organ failure, which are important causes of COVID‐19 progression and death. Therefore, elucidating the inflammatory pathways of COVID‐19 is crucial for the therapeutic strategy of COVID‐19.

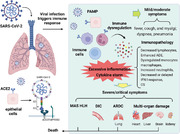

## 1 INTRODUCTION

A novel pneumonia caused by a coronavirus, named severe acute respiratory syndrome coronavirus 2 (SARS‐CoV‐2), was first reported in 2019.^1^ The novel coronavirus disease 2019 (COVID‐19) induced by SARS‐CoV‐2 has spread rapidly worldwide and has become a major global public health emergency. On March 11, 2020, the World Health Organization (WHO) declared COVID‐19 a pandemic of international concern, and as of September 2021, the impact of COVID‐19 covers 221 countries, with over 210 million confirmed cases and 4.5 million deaths reported globally.^2^ Currently, few potent vaccine candidates have been successfully developed and vaccination programmes are actively underway at the global level to combat COVID‐19.^2^ Even though some repurposed drugs, immunotherapies, and other treatment options are being applied for emergency use, particularly in critical patients, effective drugs and treatments against COVID‐19 are yet to be made available. In particular, the recent dramatic increase in the number of COVID‐19 cases, the emergence of new virus variants (Omicron B.1.1.529 spectrum, British variant Alpha B.1.1.7 spectrum, South African variant Beta B.1.351 spectrum, etc.), the high clandestinity of the virus, cross‐species jumping, and zoonosis pose a great challenge against COVID‐19.^2^, ^3^ SARS‐CoV‐2 infection can cause a wide range of clinical manifestations, mild symptoms, such as fever, cough, and myalgia; moderate symptoms, such as dyspnea, pneumonia, and local inflammation; and fatal severe or critical symptoms, with some patients experiencing complications, including impaired heart, brain, lung, liver, kidney, and coagulation system function.^4^, ^5^ According to published clinical trial data in China, approximately 20% of SARS‐CoV‐2‐infected patients eventually become severe, usually within 1–2 weeks of the onset of conventional symptoms, and severe patients often present with persistent respiratory distress and symptoms of viral pneumonia.^5^, ^6^, ^7^ Severe infection symptoms usually manifest as pneumonia, disseminated intravascular coagulation (DIC), acute respiratory distress syndrome (ARDS), hypotension, and multiorgan failure.^8^, ^9^, ^10^ Notably, in immunopathology, SARS‐CoV‐2 infection induces dysregulated innate and adaptive immune responses, causes excessive activation of inflammatory pathways and releases inflammatory factors, which are directly associated with disease severity and death.^8^, ^11^, ^12^ Excessive inflammatory responses and dysregulation of host immune defenses sustain the pathogenesis of COVID‐19, and ultimately lead to a high COVID‐19 inflammatory phase, called cytokine storm (CS), which causes deleterious tissue damage at the site of viral entry and at the systemic level.^13^, ^14^, ^15^ This phenomenon is similar to cytokine release syndrome (CRS) and secondary hemophagocytic lymphohistiocytosis.^16^

The exact pathogenesis of SARS‐CoV‐2 is not yet fully understood and combined with the novelty and high mutation rate of the virus, vaccines or effective drugs are limited in preventing or treating COVID‐19.^17^, ^18^, ^19^ It is clinically important to uncover the inflammatory pathways associated with COVID‐19 pathogenesis, including induction, function, downstream signaling, and inflammatory cytokines, for the development of more effective COVID‐19 therapeutic strategies. This article is a review that summarizes the major inflammatory pathways in the pathogenesis of COVID‐19 and highlights their potential targets and drug candidates for immune intervention.

## 2 COVID‐19 PATHOGENESIS

### 2.1 Infection of SARS‐CoV‐2

**FIGURE 1:**
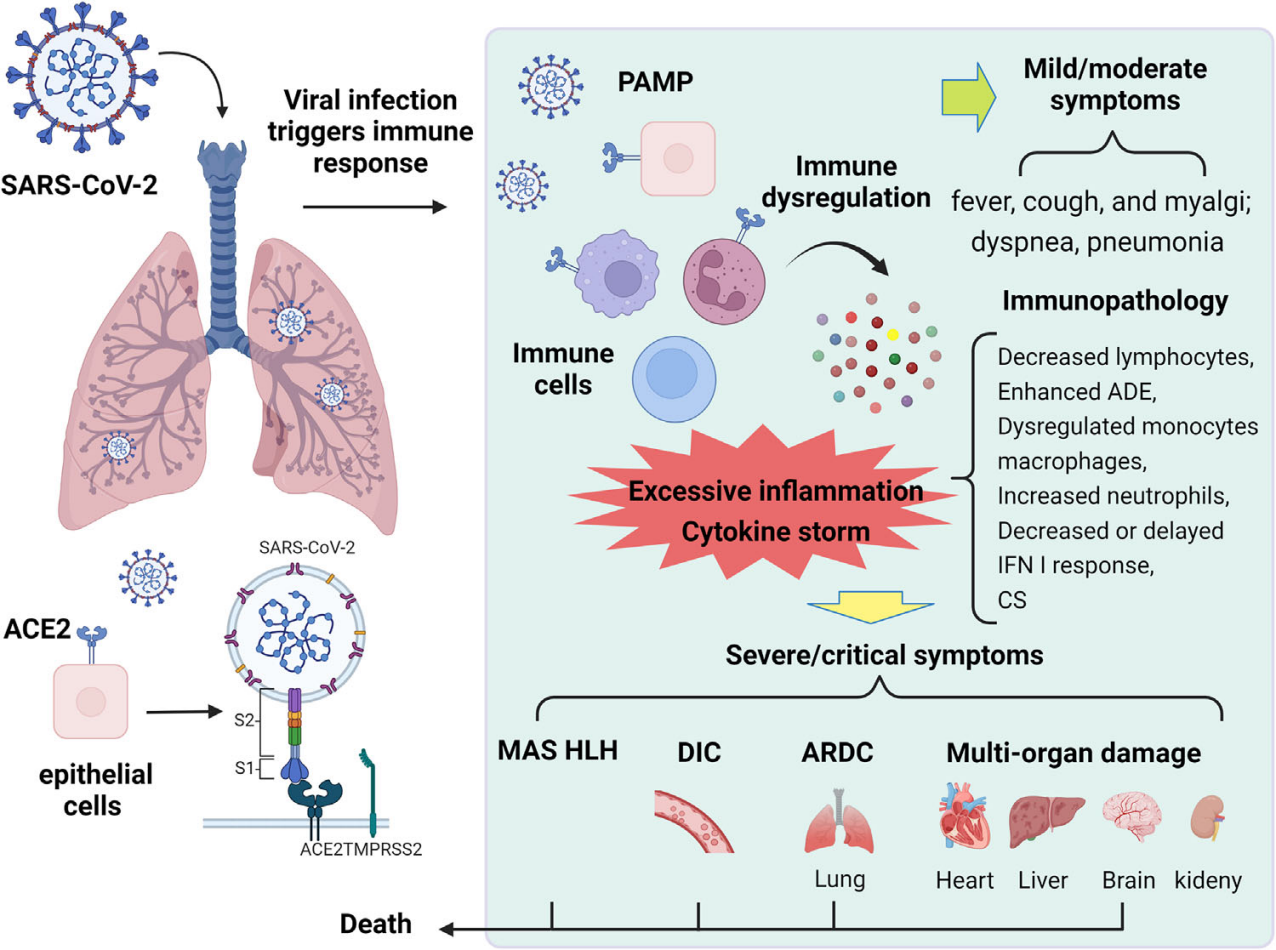
Entry of SARS‐CoV‐2 and pathogenesis of COVID‐19. SARS‐CoV‐2 enters the host and its S protein interacts with ACE2 and is initiated by TMPRSS2, which promotes endocytosis to infect respiratory epithelial cells and immune cells. During infection, rapid viral replication triggers PAMP and DAMP, causing a strong immune response and immune dysregulation. Immune cells are extensively activated and secrete large amounts of inflammatory factors, causing excessive inflammation and cytokine storm, which can lead to immunopathological impairment of COVID‐19, closely related to the severity of the disease. The clinical presentation of COVID‐19 patient progresses from mild or moderate symptoms (fever, cough, myalgia, and pneumonia) to severe or critical symptoms (MAS, HLH, ARDS, DIC, and multiorgan failure) and eventually to death. The figure is drawn by BioRender software. Abbreviations: ACE2, angiotensin‐converting enzyme 2; ARDS, acute respiratory distress syndrome; DAMP, danger‐associated molecular pattern; DIC, disseminated intravascular coagulation; HLH, hemophagocytic lymphohistiocytosis; MAS, macrophage activation syndrome; PAMP, pathogen‐associated molecular pattern; TMPRSS2, transmembrane serine protease 2

Coronaviruses (CoVs) are a highly diverse family of enveloped, justified single‐stranded RNA viruses that can infect humans, other mammals, and birds.^20^ In 2002 and 2012, two zoonotic highly pathogenic coronaviruses have emerged: the severe acute respiratory syndrome coronavirus (SARS‐CoV) and the Middle East respiratory syndrome coronavirus (MERS‐CoV).^21^ SARS‐CoV‐2 is the third zoonotic highly pathogenic coronavirus that crosses the species barrier, infects humans, and transmits between humans.^22^, ^23^ It is more transmissible than the first two, and has caused into a global pandemic. SARS‐CoV‐2 belongs to the genus Betacoronavirus, which is a member of the Coronavirinae family. Its viral particle is spherical in shape, approximately 60–140 nm in diameter, consists of RNA and phosphorylated proteins in the nucleocapsid.^24^, ^25^ SARS‐CoV‐2 encodes at least four major structural proteins, including spike protein (S), membrane protein (M), envelope protein (E), and nucleocapsid protein (N), which together with accessory proteins and other nonstructural proteins are associated with viral entry, transcription, and translation processes.^24^, ^26^, ^27^ The spike protein (S) located on the surface of SARS‐CoV‐2 acts on viral entry and is essential for the infection and pathogenesis of COVID‐19.^28^, ^29^ The pathogenesis of SARS‐CoV‐2 infection includes viral entry, binding to host cell receptors, and viral replication. Angiotensin‐converting enzyme 2 (ACE2) is a membrane surface receptor widely expressed and distributed in human tissue cells.^30^ It is abundant in lung, kidney, blood vessels, heart, liver, gastrointestinal tract, and oral mucosa with important physiological functions.^31^, ^32^ SARS‐CoV‐2 enters the host through the receptor‐binding domain (RBD) of the S protein and interacts with ACE2 on host cells, and it has a higher affinity for the ACE2 binding complex compared to SARS‐CoV (Figure 1).^26^, ^33^ In addition, the S protein of SARS‐CoV‐2 is initiated by the host cell transmembrane serine protease type 2 (TMPRSS2), which promotes virus–host cell membrane fusion and endocytosis (Figure 1).^34^ Upon entry, the virus releases its RNA genome as a template for transcription and translation, and its gene expression products include structural and nonstructural proteins that promote viral replication and pathogenesis.^35^

As an RNA virus, SARS‐CoV‐2 has a higher mutation rate than DNA viruses. From January to September 2021, several variants of concern (VOCs, Alpha, Beta, Gamma, Delta, and Omicron) of SARS‐CoV‐2 emerged and became prominent strains in many countries.^36^ In particular, the latest Omicron variant, which is the most highly mutated of the VOCs, has been designated by the WHO as one of the most dangerous VOCs. It has 50 mutations in the whole genome and 26–32 mutations in the stinger protein, and these mutations increase the tightness and affinity of RBD binding to the hACE2 receptor, which, in turn, enhances the replication capacity and infectivity of the virus.^37^, ^38^ Therefore, SARS‐CoV‐2 infection and inflammatory disease progression face new challenges, which require us to explore the interventional treatment of COVID‐19 from the perspective of controlling viral invasion and decreasing inflammation.

### 2.2 Pathogenesis of SARS‐CoV‐2 and cytokine storm

The pathogenesis of SARS‐CoV‐2 infection in human progresses from mild symptoms to severe respiratory failure, which is closely related to the immune inflammatory response. After binding to respiratory epithelial cells, the virus begins to replicate and migrate down through the airways into the alveolar epithelial cells of the lungs, and during infection, rapid viral replication may trigger a strong immune response and immune deregulation.^39^ The immunopathology of COVID‐19 is characterized by reduced lymphocytes, antibody‐dependent enhancement, dysregulated monocytes and macrophages, neutrophilia, and reduced or delayed interferon (IFN)‐I response and CS (Figure 1).

Several studies have reported that SARS‐CoV‐2 infection induces an excessive inflammatory response and CS, which can cause severe immunopathological damage and may contribute to the deterioration of COVID‐19.^13^, ^15^ CS is a rapidly progressive, life‐threatening clinical disease. It is characterized during SARS‐CoV‐2 infection by overproduction of proinflammatory cytokines and excessive activation of immune cells, which leads to persistent fever, lymphohistiocytosis (HLH), ARDS, DIC, capillary leak syndrome, multiorgan failure, and even death (Figure 1).^40^ Coronavirus infections trigger the innate immune system, which recognizes pathogen‐associated molecular patterns (PAMPs) through pathogen recognition receptors.^15^ Subsequently, multiple inflammatory pathways are activated that promotes dendritic cell maturation and macrophage activation and secretion of proinflammatory cytokines (including interleukin [IL]‐6, IL‐1β, IL‐2R, and IL‐8; tumor necrosis factor‐α [TNF‐α]; and IFN‐γ) and chemokines (C‐base sequence chemokine ligands; CCL‐2, CCL‐3, and CCL‐10).^40^, ^41^, ^42^ High viral titers and cytokine/chemokine responses recruit more innate immune cells (monocytes, macrophages, neutrophils, DCs, and NK cells) and activate adaptive immune cells (CD4^+^ and CD8^+^ T cells) from peripheral tissues, which cause hyperactivation of the immune system and massive inflammation and lead to an inflammatory CS.^11^, ^39^, ^43^, ^44^, ^45^ In severe cases, inflammatory cytokines and biomarkers, such as IL‐2, IL‐6, granulocyte colony‐stimulating factor, granulocyte‐macrophage colony‐stimulating factor (GM‐CSF), macrophage inflammatory protein 1‐α, TNF‐α, C‐reactive protein (CRP), IFNγ‐inducible protein 10 (IP‐10), and ferritin, are elevated.^8^, ^46^ These inflammatory factors exacerbate ARDS, which is the leading cause of death in patients infected with SARS‐CoV‐2.^47^, ^48^ Notably, in COVID‐19 patients, the host immune system initiates protective immune regulation to keep inflammation at appropriate levels, such as Tregs producing regulatory cytokines, such as IL‐10 and TGF‐β, to antagonize immune overstimulation. However, in viral infections leading to inflammatory attacks, such as CS, this regulatory capacity is very limited. In addition, the depletion of CD4^+^ and CD8^+^ T cells and reduction of regulatory T cells may also lead to increased inflammatory response and CS development, which, in turn, promotes viral entry to various sites and tissue damage at the systemic level and thus maintain the pathogenesis of COVID‐19.^48^ The renin‐angiotensin system (RAS) system is also involved in the pathogenesis of COVID‐19 and the progression of the CS.^49^ Viral entry into host cells downregulates ACE2 receptors, which leads to RAS dysfunction.^50^ ACE2 has an important role in pulmonary protection by catalyzing the degradation of angiotensin II (Ang II) to angiotensin (1–7) (Ang 1–7), which exerts vasoconstrictive and anti‐inflammatory effects. Ang II is a potent vasoconstrictor and has an important role in maintaining blood pressure and cell proliferation by mediating the release of proinflammatory cytokines. ACE2 downregulation leads to Ang II accumulation, which activates angiotensin II receptor 1 (AT1R) mediating lung injury, inflammation, hypokalemia, and inhibition of the pulmonary protective function of AT2R.^51^

In addition to its metabolic role, the gut microbiota also has a key role in the development and regulation of the immune system. Gut microbes coordinate immune homeostasis by producing metabolites (SCFAs), inducing proinflammatory responses (Th17 and regulatory T cells) and cytokines.^52^ Notably, the gut microbiota crosstalk with the lung and the gut microbiota affects immune functions in the respiratory epithelium, including the secretion of type I IFNs (IFN‐α and IFN‐β) and cytokines (IL‐10, TGF‐β, and IL22) to limit viral replication.^52^, ^53^ Thus, in the pathogenesis of COVID‐19, bidirectional crosstalk between the gut and the lungs (gut–lung axis) acts on the maintenance of immune homeostasis and lung health.^54^ Experimental evidence suggests that patients infected with SARS‐CoV‐2 have significantly altered lung microbiota and gut microbiota, which may be relevant to the course and severity of COVID‐19.^55^, ^56^ SARS‐CoV‐2 infection leads to pulmonary and intestinal hyperpermeability through inflammatory mediators. The ACE2 expression is even higher in the gastrointestinal tract than in the lungs, and the virus enters the circulation to bind intestinal ACE receptors and distribute them at high levels, which induces ecological dysregulation, intestinal inflammation, and gastrointestinal symptoms. Subsequently, immune system dysregulation mediates the entry of intestinal microbes, inflammatory mediator signals, and their metabolites into the circulation, which allows them to migrate to various organs (including the lung and brain) and promotes viral transmission, CS, and multiorgan damage.

Therefore, the study of targeting inflammatory pathways and cytokines from immune signaling pathways is important to guide the prevention and treatment of SARS‐CoV‐2 and its variants.

## 3 INFLAMMATORY PATHWAYS IN COVID‐19

### 3.1 Innate immune pathways activated by viral infection

SARS‐CoV‐2 enters host cells and is sensed by toll‐like receptors (TLRs), NOD‐like receptors and NLRP3, causing an IFN‐I response and its associated innate immune response. The virus promotes viral replication and severe infection through innate immune restriction mechanisms and evasion. The abnormally active innate immune response in the late stages of severe COVID‐19 induces dysregulation of downstream inflammatory pathways and massive production of proinflammatory cytokines. These cytokines continue to act on immune cell receptors and create a vicious cycle that leads to excessive inflammation and CS.

#### 3.1.1 TLR pathway

**FIGURE 2:**
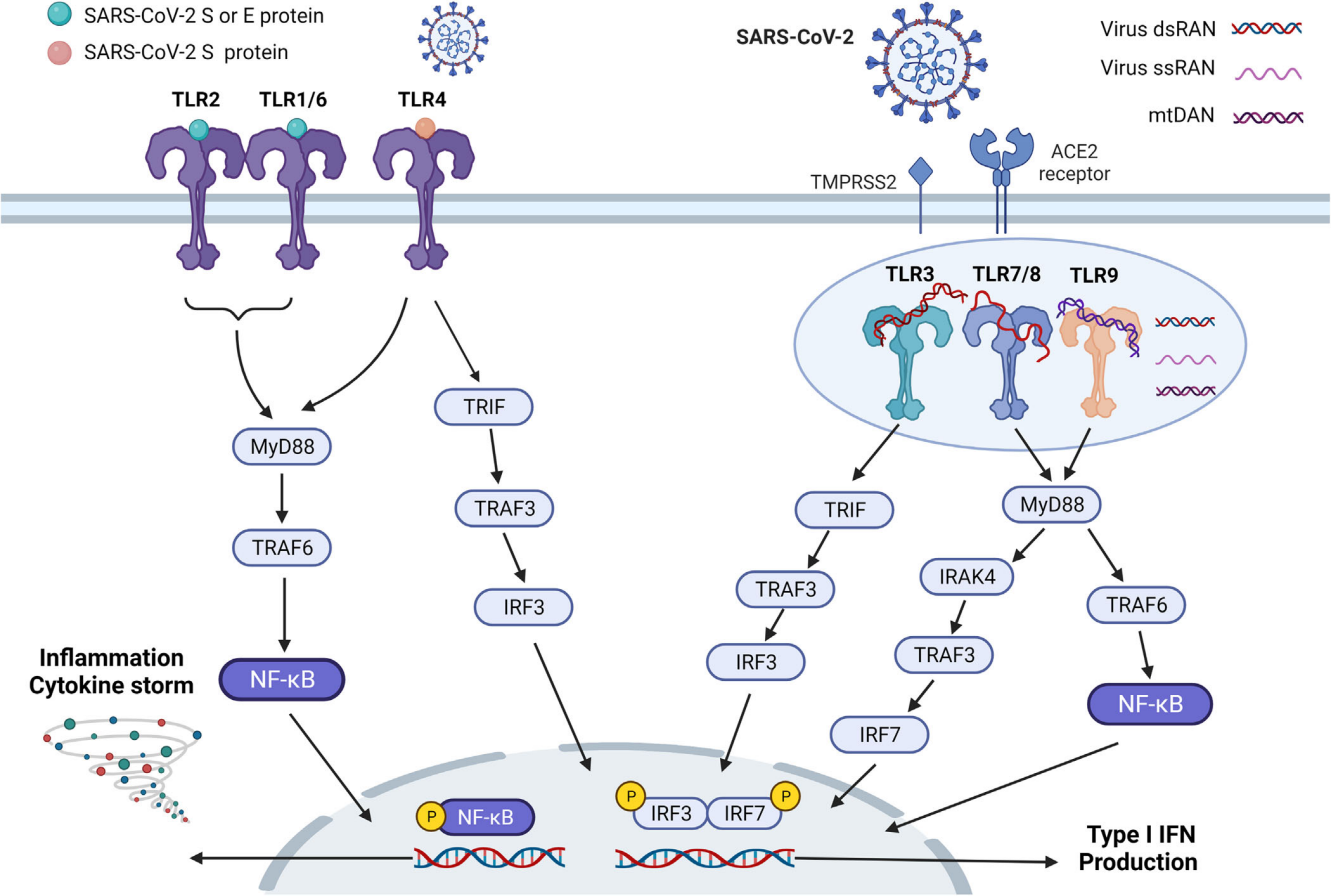
Toll‐like receptor signaling pathway in COVID‐19. TLR2/6 and TLR4 are located on the cell membrane, while TLR3, TLR7/8, and TLR9 are located on the endosome surface. TLRs recognize invading pathogens by sensing PAMP and activate the regulation of host innate immunity and cytokines. TLR activation leads to the production of proinflammatory cytokines and I IFN through its major downstream proteins MYS88 and TRIF. The S protein of SARS‐CoV‐2 activates TLR2 and TLR4; TLR3 senses intracellular viral dsRNA; TLR7/8 recognizes viral ssRNA; and TLR9 senses mtDNA damage caused by viral infection. Activated TLR regulates the production of proinflammatory factors through a series of signaling in the NF‐κB pathway and activates IRF3/7 to produce I IFN. The figure is drawn by BioRender software. Abbreviations: dsRNA, double‐strand RNA; I IFN, type 1 interferon; IRAK4, interleukin‐1 receptor‐associated kinase 4; mtDNA, mitochondrial DNA; MYD88, myeloid differentiation primary response 88; NF‐κB, nuclear factor‐kappaB; ssRNA, single‐strand RNA; TRAF, tumor necrosis factor receptor‐associated factor; TRIF, TIR‐domain‐containing adapter‐inducing interferon‐β

TLRs belong to a family of innate immune receptors that recognize invading pathogens by sensing PAMP and activate regulation of host innate immunity and cytokine expression.^57^ The TLR pathway is involved in SARS‐CoV‐2 recognition and innate immunity, and it is expressed in immune cells, fibroblasts, epithelial cells, and ACE2‐containing type II lung cells.^58^, ^59^, ^60^ Today, 11 TLR genes have been identified in humans, and 10 TLRs (TLR1–10) are expressed as receptors, TLR1, TLR2, TLR4, TLR5, TLR6, and TLR10 are expressed on the cell surface to recognize pathogen proteins and lipids; TLR3, TLR7, TLR8, and TLR9 sense pathogen nucleic acid ligands intracellularly.^61^ Activated TLRs signal through the recruitment of specific bridging molecules (myeloid differentiation primary response protein 88 [MyD88] and TIR‐domain‐containing adapter‐inducing interferon β [TRIF]), which, in turn, activate IFN regulatory factor (IRF, IRF‐3, and IRF‐7) and nuclear factor kappa‐B (NF‐Κb) pathways to induce Type I IFN and inflammatory cytokine production (Figure 2). TLR4 signals through the MyD88 and TRIF pathways, TLR3 signals through TRIF, and the other TLRs are mediated through the MyD88 pathway. Currently, TLR2, TLR3, TLR4, TLR7, TLR8, and TLR9 are considered as possible receptors involved in SARS‐CoV‐2 infection and correlate with disease severity (Figure 2).^62^ TLR‐mediated immune dysregulation is critical for COVID‐19 hyperinflammation and CS, and is a target for COVID‐19 intervention therapy (reviewed in Refs. 63, 64, 65).

SARS‐CoV‐2 S and E proteins are identified as ligands for TLR2, which forms heterodimers with TLR1 and TLR6 for signaling through MyD88 and TRIF.^62^, ^66^ TLR2 mediates activation of the NF‐κB pathway to induce inflammatory mediators in macrophages, monocytes, and human lung epithelial cells. A computer study suggested that SARS‐CoV‐2 S protein may bind strongly to TLR4 and two subsequent studies demonstrated the binding site of S protein to TLR4.^67^, ^68^, ^69^ The S1 subunit of the virus (residues 16–671) induces activation of NF‐κB and MAPK pathways, and the N‐terminal structural domain (residues 1–307) or RBD (residues 319–541) may be associated with activation of the immune response in macrophages. In addition, the activation of TLR4 may increase ACE2 expression, NETosis, and inflammasome activation, facilitating viral entry and causing excessive inflammation.^70^, ^71^, ^72^, ^73^

TLR3 senses intracellular viral dsRNA and leads to the production of proinflammatory cytokines and type I IFN through the activation of TRIF and its downstream signaling pathways (IRF3 and NF‐κB).^74^ TLR3‐mediated overactivation of dsRNA may lead to cardiovascular complications, such as vascular inflammation and hypertension. A study characterized by the SARS‐CoV‐2‐associated molecular model (SAMP) identified the TLR7/8/MyD88 pathway is a key pathway for activation of human pDC and cDC, and is involved in inflammation and immune activation.^75^ TLR7/8 recognize two GU‐rich short sequences in the ssRNA genome of SARS‐COV‐2 and activate the MyD88 pathway, with TLR7 involved more in the antiviral immune response and TLR8 controlling the production of proinflammatory cytokines. SARS‐CoV‐2 infection damages mitochondrial function to release mtDNA and activate TLR9/MyD88 signaling in endothelial cells, thereby triggering inflammatory responses and endothelial cell dysfunction.^76^, ^77^ Therefore, regulation of the TLR pathway is important for COVID‐19‐activated immunity, and targeting the TLR pathway is ideal for blocking viral transmission and excessive inflammation.

#### 3.1.2 NLRP3/IL‐1β pathway

The Nod‐like receptor family, pyrin domain‐containing 3 (NLRP3), belongs to the NOD‐like receptor family. In general, activation of the NLRP3 inflammasome requires two steps, initiation and activation. Possible mechanisms for activation of the NLRP3 inflammasome by SARS‐CoV‐2: the first step activates (initiates) the TLR/NF‐κB pathway; the second step (activation) includes E protein and ORF3a protein‐mediated ion transport (Ca^2+^ and K^+^), S protein‐mediated accumulation of Ang II, N protein‐mediated complement cascade, and the direct binding of ORF8b to the LRR structural domain of NLRP3 (reviewed in Ref. 78). SARS‐CoV‐2 drives the assembly of the NLRP3 inflammasome of the proinflammatory protein complex (reviewed in Refs. 79, 80, 81, 82). The complex consists of a sensor (NLRP3), an adaptor (ASC), and an effector (caspase 1), which acts on the processing of cytokines (IL‐1 and IL‐18).^83^ IL‐1β is a representative member of the IL‐1 family that is closely associated with inflammation and has been extensively studied. IL‐1β production is formed by cleavage of inactivated IL‐1β precursors by the NLRP3 inflammasome, which binds to the IL‐1 receptor, activates the intracellular NF‐κB cascade response, and creates a positive feedback on its own production.^84^ IL‐1β formation and its signaling facilitate the migration of immune cells to inflammatory tissues, Th17 cell differentiation, PAMP/DAMP generation, and the expression and release of various cytokines, which has a positive effect on the pathogenesis of COVID‐19 and CS.^85^ Activation of the NLRP3 inflammasome also leads to a proinflammatory form of programmed cell death called cytokinesis.^86^ The results are explained by the fact that antibody‐mediated SARS‐CoV‐2 infection of monocytes/macrophages activates inflammation and cytokine release and causes severe COVID‐19 pathogenesis. Therefore, inhibition of NLRP3 and its signaling, or direct blockade of IL‐1 becomes a potential therapy strategy for COVID‐19.

### 3.2 JAK/STAT signaling pathway

**FIGURE 3:**
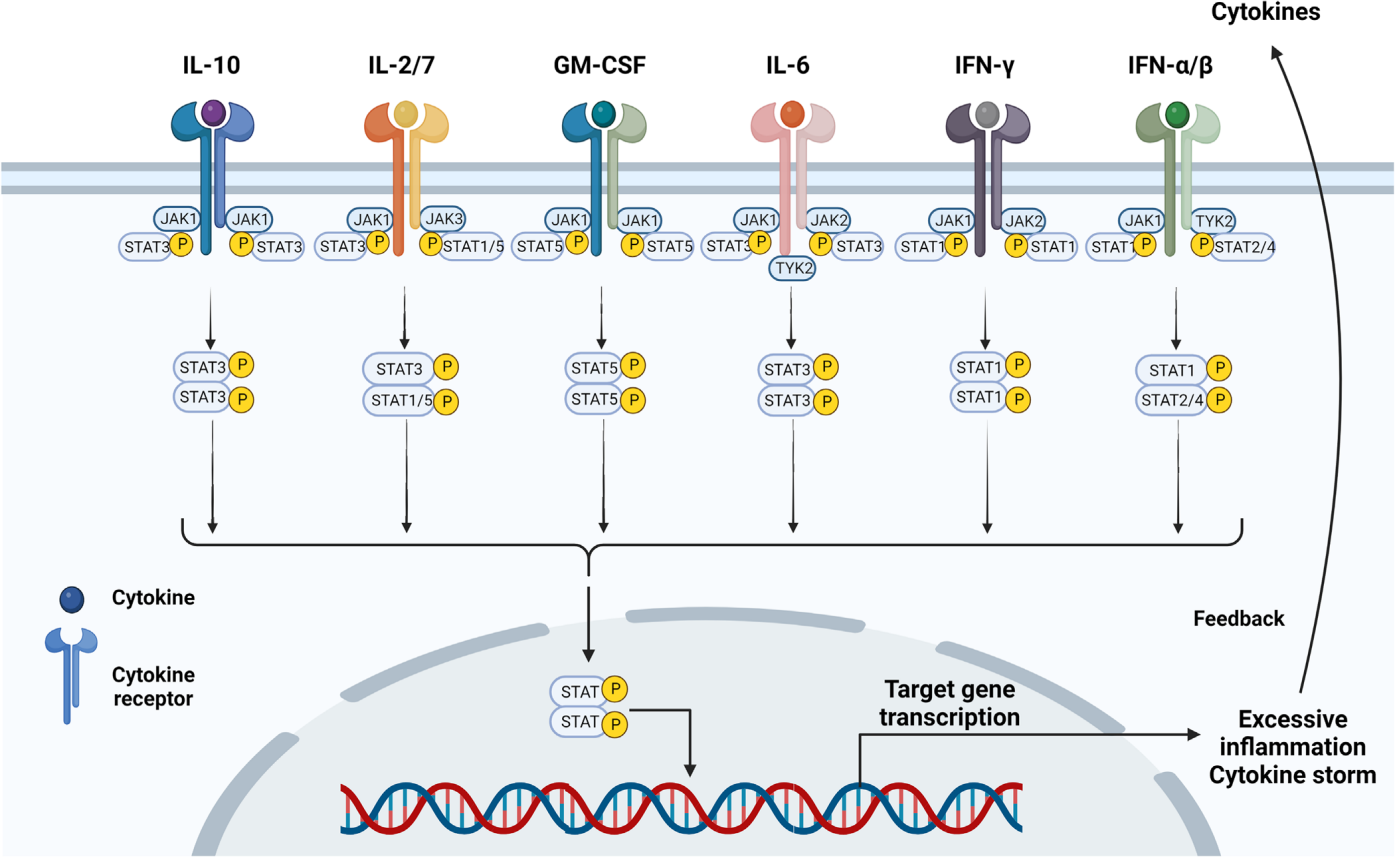
JAK/STAT pathway in COVID‐19. JAK transmits extracellular signals for many inflammatory factors (IL‐2, IL‐4, IL‐6, IL‐7, TNF‐α, IL‐10, GM‐CSF, and IFN), JAK phosphorylates and subsequently activates STAT dimers, which translocate the signals into the nucleus. These inflammatory factors promote inflammation, cytokine storm, and COVID‐19 pathogenesis through the JAK/STAT pathway. The figure is drawn by BioRender software.

The JAK/STAT pathway is the main pathway involved in COVID‐19 inflammation and plays a key role in the course of the CS.^87^, ^88^ Janus kinase (JAK) is a family of tyrosine kinases associated with cytokine receptors in the cytoplasm, which consists mainly of JAK1, JAK2, JAK3, and tyrosine kinase 2 (TYK2). JAK transmits extracellular signals from many proinflammatory factors to activate signal transducers and activators of transcription (STATs) and together with it constitutes one of the major cellular signaling pathways (JAK/STAT) in the immune response.^89^, ^90^ The STAT protein family consists mainly of STAT1, STAT2, STAT3, STAT4, STAT5a, STAT5b, and STAT6, which contains structural domains that bind to proteins and DNA. The initiation of the JAK/STAT pathway is mediated by high–affinity interactions between many different extracellular signals (growth factors, cytokines, and hormones) and their cognate receptors.^91^ The JAK nonreceptor tyrosine kinase receives the extracellular signal and the associated receptor undergoes a conformational change, JAK tyrosine phosphorylation, and subsequent activation of STAT dimerization, which transduces the signal into the nucleus (Figure 3). STAT dimers bind to specific DNA in the nucleus and regulate the transcription of genes related to immune response, apoptosis, cell cycle, proliferation, and differentiation.^92^ Severe COVID‐19 patients have elevated levels of inflammatory cytokines, including IL‐2, IL‐4, IL‐6, IL‐7, TNF‐α, IL‐10, GM‐CSF, and IFN, which may be associated with CS, many of them regulate the immune response and inflammation via the JAK/STAT pathway (Figure 3).^8^, ^93^, ^94^ IL‐2 is mainly produced by CD4^+^ T cells and regulates the activation and proliferation of CD4^+^, CD8^+^ T cells, NK, and other immune cells. It involves in the regulation of physiological and pathological processes, such as immune responses through the IL‐2/IL‐2R/JAK/STAT5 signaling pathway, and is used clinically in the treatment of viral infections, autoimmune diseases, and tumors.^95^, ^96^ IL‐7 acts in a similar manner to IL‐2. One study showed that IL‐2 was elevated in general and severe patients but decreased in patients with critical COVID‐19 pneumonia. This may be due to the suppression of the IL‐2/IL‐2R/JAK/STAT5 pathway by reduced levels of IL‐2, IL‐2R, JAK1, and STAT5, leading to a decrease in lymphocytes (CD8^+^ and CD4^+^ T cells) in critical COVID‐19 pneumonia patients.^97^ IL‐10 is secreted in a variety of immune cells, including Th2 cells, CD8^+^ T cells, Tregs, DCs, macrophages, and NK cells, and signals through the IL‐10R/JAK1‐TYK2/STAT3 pathway.^98^ IL‐10 is an important immunomodulatory factor with anti‐inflammatory and immunosuppressive effects. It limits the innate immunity of macrophages, DC cells, exerts anti‐inflammatory effects indirectly through Tregs, and enhances the function of mast cells, CD8^+^ T, B, and NK cells.^99^ Although the overproduction of IL‐10 may act as a negative feedback regulator to antagonize an overactive immune system, the anti‐inflammatory ability of IL‐10 is very limited in a state of massive secretion of inflammatory mediators and extensive activation of inflammatory cells in COVID‐CS.^100^

#### 3.2.1 IL‐6/JAK/STAT pathway in COVID‐19

IL‐6 is mainly produced by a variety of cells, including immune cells, such as B cells, T cells, macrophages, DCs, monocytes, and nonimmune cells, such as fibroblasts and endothelial cells, especially bronchial epithelial cells. IL‐6 is expressed at high levels in COVID‐19 patients and is strongly correlated with disease severity and is one of the most important inflammatory markers of COVID‐19, which can be used to predict and diagnose hyperinflammation and CS.^8^, ^15^, ^101^, ^102^ It plays a key role in immune pathogenesis, contributing to immune cell hyperactivation and target organ dysfunction in CS, and is associated with CRS, ARDS, and poor clinical outcomes in COVID‐19.^103^, ^104^ Its hematopoietic and platelet‐producing effects are also used as a diagnostic tool to assess the severity of inflammation. In addition to hepatocytes, IL‐6 promotes the secretion of acute phase proteins, such as CRP, iron‐regulating hormone, thrombopoietin, complement C3, and ferritin, which play a role in hypoferritinemia associated with chronic inflammation.^105^, ^106^

In the immune response to COVID‐19, IL‐6 is mainly involved in two JAK/STAT pathways: the classical cis‐signaling pathway and the trans‐signaling pathway. IL‐6R exists in transmembrane form (mIL‐6R) and soluble form (sIL‐6R), mIL‐6R is mainly expressed on immune cells and sIL‐6R is expressed on almost all cell types. IL‐6 binds to both forms of the receptor and then interacts with gp130 to trigger downstream JAK/STAT3 signaling and gene expression.^107^, ^108^ In the classical signaling pathway, IL‐6 binds to mIL‐6R and then forms an IL‐6/IL‐6R/gp130 complex with the membrane protein gp130 to initiate downstream intracellular signaling of JAK/STAT3. Activation of this pathway has multiple effects on the immune response, promoting the differentiation of Th17, CD8^+^ T and B cells; enhancing neutrophil migration; and reducing Tregs, further promoting IL‐6 secretion, exacerbating inflammation, and causing CS. In trans‐signaling, high concentrations of circulating IL‐6 bind to soluble forms of IL‐6R (sIL‐6R) and form complexes with gp130 dimers in cells that do not express mIL‐6R, such as endothelial cells, activating IL‐6/sIL‐6R/JAK/STAT3 signaling. Activation of this signaling pathway promotes the secretion of multiple inflammatory factors, including vascular endothelial growth factor (VEGF), monocyte chemotactic protein‐1 (MCP‐1), IL‐8, and more IL‐6, leading to a systemic “cytokine storm.”^108^, ^109^ Dysregulation of the IL‐6 inflammatory pathway also promotes atherosclerosis and enhances vascular permeability, acting on cardiovascular disease in COVID‐CS, hypotension, and pulmonary dysfunction in ARDS.^104^, ^110^, ^111^ The IL‐6/JAK/STAT signaling pathway plays an important role in proinflammatory cytokine overexpression, viral infection, and pathogenicity, and may be a key therapeutic target for COVID‐19.

#### 3.2.2 IFN/JAK/STAT pathway in COVID‐19

IFN is divided into two types according to its effects: type I IFN includes IFN‐α and IFN‐β, which can be secreted by human leukocytes and fibroblasts and mainly exerts antiviral effects; type II IFN includes IFN‐γ, which can be secreted by immune cells, such as T cells and NK cells, and mainly exerts immunomodulatory effects. In addition, there is a third type of IFN, IFN‐λ, which acts similar to type I IFNs, but the unique characteristics of the IFN‐λ response are its concentration, persistence, and noninflammatory nature.^112^ IFN‐λ is mainly produced by epithelial cells of the respiratory and gastrointestinal tracts, acts on IFN‐λR (IL28Rα and IL‐10Rβ) on epithelial cells and neutrophils to inhibit viral replication without causing inflammation.^113^, ^114^ An increasing number of clinical studies show that COVID‐19 patients have high levels of proinflammatory cytokines and low levels of type I and III IFNs in their serum; protective IFN‐I responses are significantly reduced or delayed in severe COVID‐19 patients.^44^, ^115^, ^116^ However, some studies have reported elevated levels of IFN‐γ in COVID‐19 patients, presumably contributing to COVID‐CS.^8^, ^117^ Furthermore, a delayed IFN‐I response not only hinders viral clearance but also induces excessive inflammation and exacerbates the immunopathological damage in COVID‐19 patients.^118^ During coronavirus infection, the steps of IFN regulation of the innate antiviral immune response are (1) innate recognition, (2) IFN production, (3) IFN signaling, and (4) interferon‐stimulated gene (ISG) effectors. The signal transduction of IFNs is mainly through the JAK/STAT pathway, which, in turn, upregulates various genes controlled by IFN.

The IFN‐I response is the first line of protective response of the host immune system, counteracting immunity and excessive inflammation by promoting viral clearance and modulating innate and adaptive immune responses. IFN‐I activates the JAK1/TYK2/STAT1/2 pathway and promotes the formation of the STAT1/2/interferon regulatory factor 9 complex, which, in turn, regulates ISG transcription to produce antiviral mediators.^119^ Type III IFNs share the same signaling pathway as type I IFNs, but IFN‐λs result in more sustained ISG expression and may provide more durable antiviral protection in the upper respiratory tract, which limits viral transmission.^119^, ^120^ SARS‐CoV‐2, similar to SARS‐CoV and MERS‐CoV, presumably already possesses mechanisms to avoid immune recognition and inhibit IFN and ISG function.^121^, ^122^ This defense mechanism may, therefore, serve as a key factor in antiviral resistance and the use of IFN or JAK inhibitors as a prospect and challenge for COVID‐19 treatment.

#### 3.2.3 Angiotensin II and AT1R signaling in the JAK/STAT pathway

The RAS involves in the development of SARS‐CoV‐2 infection and COVID‐19‐CS, the pathogenesis of which is ACE2 receptor shedding leading to RAS dysfunction.^49^ Angiotensin II (Ang II) binds to angiotensin II type 1 (AT1) receptors on the surface of different cells and activates the JAK2/STAT (STAT1/2/3) pathway based on target cells, in turn promoting the local production of proinflammatory cytokines, such as IL‐6, IFN‐γ, and TNF‐α.^123^, ^124^, ^125^

#### 3.2.4 GM‐CSF signaling and the JAK/STAT pathway

GM‐CSF is a hematopoietic growth factor that is expressed in small amounts in the alveoli, but in the inflammatory state of COVID‐19 infection, dysregulation of GM‐CSF results from the activation of immune cells, such as leukocytes, DCs, Th17 cells, and inflammatory factors.^93^, ^126^

High levels of GM‐CSF expression result in significant recruitment and activation of myeloid cells, leading to the massive release of proinflammatory mediators, excessive systemic inflammation, and lung injury.^127^, ^128^ This is particularly seen in patients with ADRS, CRS, and severe COVID‐19. GM‐CSF activates the JAK2/STAT3/STAT5 signaling pathway by binding to the β‐chain subunit of the GM‐CSF receptor, thereby regulating the massive release of cytokines, such as IL‐1, IL‐6, and TNF‐α from immune cells (such as DCs, monocytes, and macrophages).^129^, ^130^ In addition, GM‐CSF‐R may activate other downstream signals, including the activation of NF‐κB and PI3K‐Akt.^131^ Thus, regulation of GM‐CSF and its signaling is an important way to improve inflammation in COVID‐19.

### 3.3 NF‐κB signaling pathway

**FIGURE 4:**
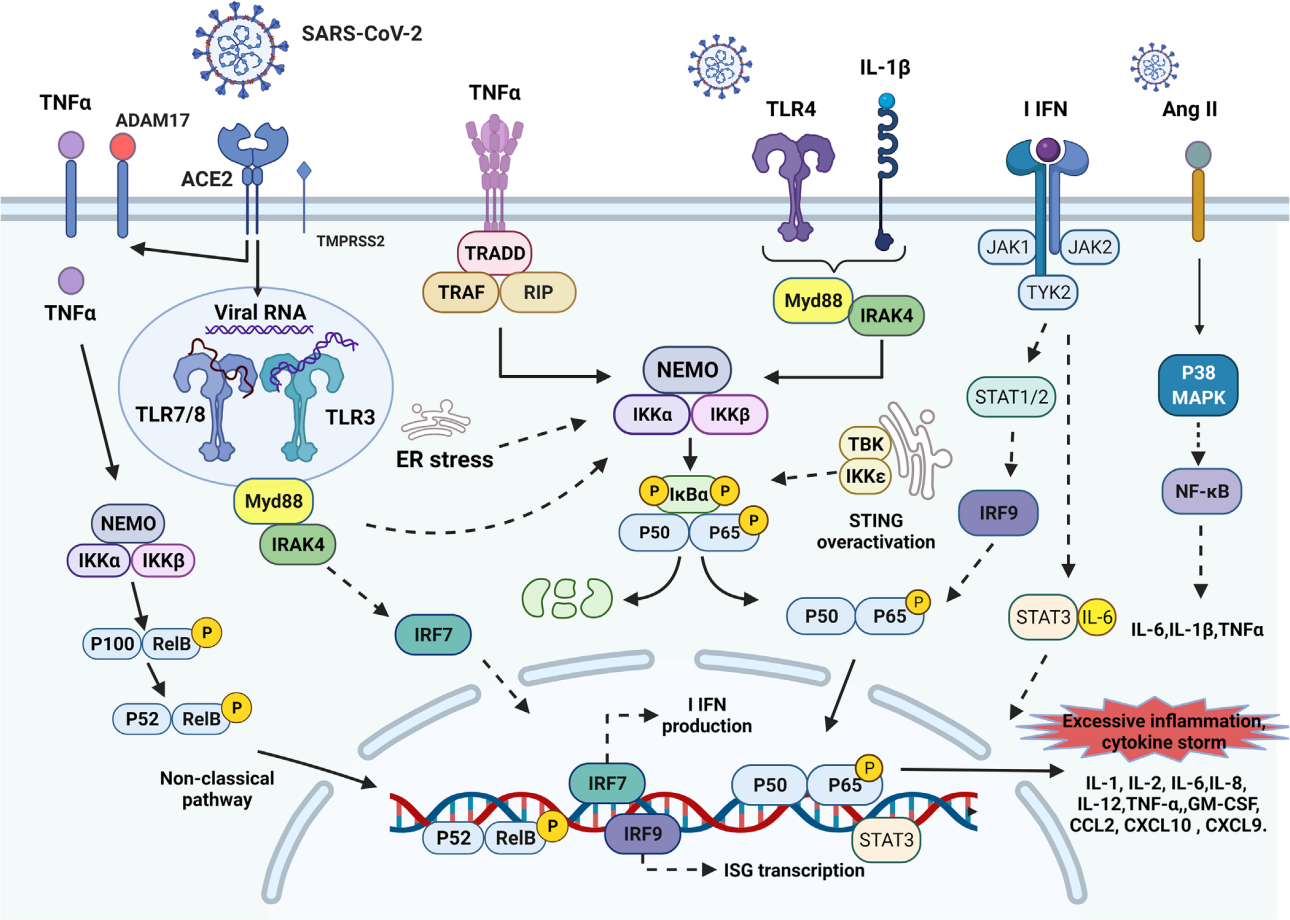
The NF‐κB pathway and its crosstalk cellular signaling pathway in COVID‐19. Activation of the classical pathway involves mainly stimulation of TNF‐α, IL‐1, and Toll‐like receptor ligands, induces IκBα phosphorylation (catalyzed by IκB kinase [IKK]: IKKα, IKKβ, and NEMO complex), and in turn translocates heterodimers of p65 and p50 into the nucleus. The nonclassical pathway is stimulated mainly by specific TNF family cytokines, and IKKα‐derived p100 phosphorylation (occurs in a RelB‐dependent manner) generates the transcriptionally active p52‐RelB complex, which activates the pathway. The overactivated NF‐κB pathway produces large amounts of proinflammatory cytokines (IL‐1, IL‐2, IL‐6, IL‐8, IL‐12, TNF‐α, and GM‐CSF) and chemokines (CCL2, CXCL10, and CXCL9). This is an important pathogenic mechanism leading to COVID‐19 inflammation and cytokine storm. In addition, JAK/STAT pathway, Ang II accumulation, p38 MAPK pathway, endoplasmic reticulum stress, and STING also crosstalk with NF‐κB pathway. The figure is drawn by BioRender software.

NF‐κB is a family of highly conserved transcription factors containing five proteins, including the Rel‐like structural domains p65 (RelA), RelB, c‐Rel, p105/p50 (NF‐κB1), and p100/52 (NF‐κB2), which bind to each other to form homo‐ or heterodimeric complexes and have different transcriptional activities.^132^ The p50/65 heterodimer is a representative of Rel dimers and exists in almost all cell types. NF‐κB activation and function are regulated by the NF‐κB inhibitory protein (IκB) family, including IκBα, IκBβ, IκBγ, IκBε, and Bcl‐3, with IκBα being widely studied as a characteristic protein.^132^ The NF‐κB signaling pathway proceeds as follows. Activation of IκB kinase (IKK) complex (IKKα, IKKβ, and NEMO) by NF‐κB inducers (TNF‐α, IL‐1β, viral RNA, and ROS) leads to IκB phosphorylation and promotes the release of p50 and p65 dimers and nuclear translocation of NF‐κB.^133^ NF‐κB binds to specific DNA in the nucleus and activates the transcription of multiple genes related to host immunity, inflammation, cell proliferation, and apoptosis.^134^ SARS‐COV‐2 proliferates in host cells, producing and accumulating transcriptional intermediates dsRNA. Interferon‐induced dsRNA‐dependent protein kinase (PKR) production and innate immunity; second, PKR activates IKK and induces the NF‐κB classical pathway; in addition, PKR upregulates TNF‐α expression, ultimately leading to activation of the nonclassical pathway (Figure 4).^135^ Various proteins of SARS‐CoV‐2 (N, M, ORF7a, ORF3a, Nsp5, and Nsp14) induce hyperactivation of NF‐κB signaling pathway.^136^, ^137^, ^138^

The NF‐κB signaling pathway is a central player and potential therapeutic target in severe COVID‐19 and CS, whose activation promotes the expression of a large number of inflammatory factors, including cytokines (IL‐1, IL‐2, IL‐6, IL‐12, TNF‐α, and GM‐CSF) and chemokines (CCL2 [MCP‐1], CXCL10 [IP‐10], CXCL9 [MIG], and IL‐8).^139^, ^140^ Recent studies have shown that NF‐κB signaling pathway induces Activin A/FLRG to have an important role in the pathogenesis of ARDS.^141^

#### 3.3.1 TNF‐α/NF‐κB signaling pathway

TNF‐α is mainly produced by monocytes, macrophages, and T cells and is rapidly released in response to tissue injury or infection, which is strongly associated with inflammation, autoimmune diseases, and cancer. TNF‐α activates the NF‐κB signaling pathway and induces the expression of several proinflammatory and antiapoptotic genes through its receptor TNFR1 and a series of transduction processes (mediated by IKK, IκB, and NF‐κB dimers).^142^ Previous studies have demonstrated that SARS‐CoV spike protein induces substantial protein release of IL‐6 and TNF‐α, which is dependent on the activation of NF‐κB signaling pathway.^143^ However, the role of TNF‐α in COVID‐19 is still not fully understood. Some studies have reported elevated levels of TNFα in severe COVID‐19 cases and TNF‐α levels may be negatively correlated with T‐cell counts,^8^, ^48^, ^144^ while others have shown normal TNF‐α levels.^145^ A lot of evidence suggests that inhibition of TNF‐α blocking its downstream NF‐κB signaling may have a protective effect on COVID‐19.^146^, ^147^

#### 3.3.2 Crosstalk of NF‐κB pathway by other pathways

Activation and function of NF‐κB pathway crosstalk with multiple intracellular signaling pathways, which act together to promote inflammation and CS in COIVD‐19 (Figure 4). IL‐1β is processed by NLRP3 into a secretable protein that binds to IL‐1R and thus activates the NF‐κB pathway to amplify the proinflammatory effect (see Section 3.1.2 for details). During SARS‐CoV‐2 infection, multiple pathways of the NF‐κB pathway are shared with TLR‐mediated signaling cascades. TLR7/8 and TLR3 activate NF‐κB pathway signaling through a series of intermediates that induce derived IKK, TLR4 stimulates NF‐κB activation in crosstalk with the IL‐1, TNF‐α pathway, and it also activates the NF‐κB pathway through ER stress (reviewed in Ref. 140). In the intercrosstalk between the JAK/STAT3 pathway and NF‐κB, STAT3 is necessary for full activation of NF‐κB. IL‐6 mediates signaling of the JAK/STAT3 pathway interacting with the NF‐κB pathway to induce IL‐6 amplifier responses. MAPKs are one of the major cellular signaling pathways that promote NF‐κB hyperactivation. The P38‐MAPK crosstalk with NF‐κB activates the RAS system, leading to the overproduction of Ang II.^148^ Ang II acts as a proinflammatory factor, inducing NF‐κB, deintegrin, and metalloproteinase 17 (ADAM17), which together promote TNF‐α, IL‐1β, MMP‐9, and epidermal growth factor receptor to act on inflammation and CS.^149^, ^150^, ^151^, ^152^

Virus–host cell interactions induce ER stress and ER stress signaling protein (IRE‐1α)/UPR signaling. IRE‐1α is associated with NF‐κB activation, and viruses promote self‐replication and inflammation through the interplay of IRE‐1α/UPR, NF‐κB, and MAPK pathways.^153^ In addition, COVID‐19 induces dysregulation of STING (stimulator of IFN gene), which produces IFN‐β by activating IRF 3 or mediates the TBK/IKKε/NF‐κB pathway.^154^, ^155^ A review has summarized the role of crosstalk between NF‐κb and Nrf2‐Keap1 pathways on the neurological complications of COVID‐19.^156^

### 3.4 BTK signaling pathway

Bruton's tyrosine kinase (BTK), a nonreceptor tyrosine kinase is expressed primarily in myeloid and B cells and functions downstream of the receptor. During BCR signaling, the BCR cross‐links with antigen to activate phosphorylation of nonreceptor protein tyrosine kinases (Syk, Lyn, and Fyn) and its intracellular sequence (called ITAM).^157^ BTK is phosphorylated by kinase Lyn or Syk at Y551 and subsequently fully activated by autophosphorylation at Y223.^157^, ^158^ BTK interacts with PIP3 through its PH structural domain and is subsequently recruited to the plasma membrane and acts on PLCγ2 and PI3K to activate the regulation of gene transcription and calcium flux.^157^, ^158^ BTK involves in triggering receptors expressed on myeloid cells and plays an important role, which may be relevant in the pathogenesis of infectious complications, such as CD354 and TREM1 signaling in monocytes and regulation of their function and maturation in neutrophils.^159^, ^160^ Activation of the BTK–SYK axis culminates in PI3K, NF‐κB, and MAPK‐dependent downstream signaling cascades to regulate a variety of cellular processes, such as cell proliferation, differentiation, and cytoskeletal remodeling, exhibiting therapeutic potential in immunotherapy, such as rheumatoid arthritis (RA).^161^, ^162^ Although the specific mechanisms and pathways are not clear, the use of BTK inhibitors (BTKis) for the treatment of COVID‐19 results in a significant reduction of proinflammatory factors, such as IL‐6, IL‐1, TNFα, as well as relief of excessive inflammatory responses, ARDS, and thrombosis in the lung (reviewed in Refs. 163 and 164).

### 3.5 MAPK signaling pathway

Intracellular signaling pathways mediated by mitogen‐activated protein kinases (MAPKs) play important roles in a variety of cellular processes. In mammals, the MAPKs family mainly includes extracellular signal‐regulated kinases, c‐Jun N‐terminal kinases, and p38 MAPKs.^165^ The MAPK cascade response is initiated by specific extracellular signals and undergoes sequential activation of MAPK kinase kinase (MAPKKK) and MAPK kinase (MAPKK), ultimately inducing the activation of specific MAPK. The p38 MAPK pathway has been associated not only with SARS‐CoV infection but also with inflammatory injury in the lung and heart of COVID‐19 patients.^166^, ^167^

#### 3.5.1 P38 MAPK pathway

The p38 MAPK pathway is strongly activated mainly by environmental stress and inflammatory cytokines and significantly affects immune responses and inflammatory processes.^168^ The p38 family members include p38α, p38β, p38γ, and p38δ, which have different expression patterns.^169^ P38α and p38β are present in almost all cells, whereas p38γ and p38δ are more tissue specific. A variety of extracellular stimuli may induce the p38 MAPK pathway, including cellular stress, G‐coupled protein receptor, inflammatory cytokines, growth factors, IL‐1, and TGFβ; the MAPKKKs of the p38 module include MEKK, MLK2, MLK3, ASK, TAK1, TAO1, and TAO2; MAPKKs are mainly MKK3 and MKK6.^165^, ^168^ Extracellular stimulation activates the phosphorylation of p38MAPKKK, which directly activates MKK3/6, which, in turn, activates p38 MAPK and downstream substrates, such as MK2/3 kinase, PRAK, MSK1/2, and various transcription factors (ATF1/2/6 and p53).^169^ According to previous studies on SARS‐CoV viruses, p38 MAPK activation is associated with endocytosis of ACE2 and regulation of the viral life cycle.^170^, ^171^ Activation of the p38 MAPK pathway increases the production of proinflammatory factors (IL‐6, IL‐1β, and TNF‐α), which facilitates excessive inflammation and CS mediated by SARS‐CoV‐2 infection.^169^, ^172^ A number of studies have shown that SARS‐CoV‐2 promotes self‐replication, macrophage syndrome, and abnormal inflammation by regulating the p38 MAPK pathway through multiple mechanisms.^166^, ^173^, ^174^ The most notable mechanism is that SARS‐CoV‐2 infection leads to ACE2 downregulation, RAS dysregulation, and Ang II accumulation. Ang II activation of p38 MAPK induces a host Th17 response and upregulates ADAM17 to cleave the extracellular structural domain of ACE2 further reducing local ACE2 protective activity, which ultimately induces myocarditis and ARDS.^166^, ^175^, ^176^ This is in crosstalk with the NF‐κB pathway. P38 MAPK is one of the factors mediating NF‐κB signaling activation, and SARS‐CoV‐2 spike protein activates NF‐κB and AP‐1/c‐Fos through MAPK to promote IL‐6 release.^177^, ^178^ In addition, SARS‐CoV‐2 overactivates p38 MAPK as well as NF‐κB pathway by downregulating dual specificity phosphatase (DUSP), which facilitates the release of proinflammatory factors.^179^ P38 MAPK is able to affect the expression of various mi/lncRNAs and associated transcription factors, which are important for sterile inflammation and macrophage maturation, proinflammatory programming, and M2/Th2 polarization in COVID‐19 patients.^174^ Recent studies have found that SARS‐CoV‐2 can promote IL‐1β production and mediate endothelial inflammation by activating the p38 MAPK/NF‐κB pathway via ACE2 or TLR4.^180^ The p38 MAPK pathway is an important factor in mediating lung and heart injury in COVID‐19, and it promotes inflammation and CS development. Therefore, p38 MAPK inhibitors are a potential approach for the treatment of SARS‐CoV‐2 infection.

### 3.6 P13/Akt/mTOR signaling pathway

The PI3K/Akt/mTOR pathway is an important cellular signaling pathway involved in the regulation of various cellular functions, and it plays a key role in the pathogenesis of tumors, autoimmunity, and viral infections.^181^, ^182^, ^183^ The pathway is a signaling cascade process.^182^, ^184^, ^185^, ^186^ Phosphatidylinositol 3‐kinase (PI3K) is stimulated, phosphorylated to phosphatidylinositol 4, 5‐bisphosphate (PIP 2), and converted to phosphatidylinositol 3, 4, 5‐trisphosphate (PIP3). PIP3 recruits phosphatidylinositol‐dependent kinase 1 (PDK1), which activates protein kinase B (Akt). Subsequent Akt phosphorylation activates the mammalian target of rapamycin complex 1 (mTOR1), and mTORC2 can promote cell survival by activating Akt. mTOR activates its downstream effectors (4EBP1 and P70S6) and ultimately induces RNA translation, protein synthesis, and many other biological functions.^186^

A lot of evidence suggests the potential contribution of PI3K/Akt/mTOR in COVID‐19. It has been thought that the pathway of SARS‐CoV‐2 entry into cells may be regulated by PI3K/AKT signaling.^187^, ^188^ Upregulation of PI3, Akt, and mToR was detected in proteomic analyses on SARS‐CoV‐2.^189^ A protein transcriptomic study also demonstrated that SARS‐CoV‐2 infection not only improves viral replication and survival through PI3K/Akt/mTOR signaling, but also causes dysregulation of the Akt/mTOR/HIF‐1 signaling cascade.^190^ A recent article showed that SARS‐CoV‐2 induces the production of proinflammatory cytokines (IL‐8) through PI3K/Akt/mTOR pathway, which promotes the development of severe COVID‐19 and CS.^191^ Another article presented similar evidence that SARS‐CoV‐2 infection upregulates the proinflammatory chemokines CXCL9, CXCL10, and CXCL11 in an AKT‐dependent pathway.^192^ In addition, hypoxia may induce the Warburg effect in pulmonary endothelial cells, leading to vasoconstriction and thrombosis. The Warburg effect promotes SARS‐CoV‐2 replication and associated inflammatory responses, in which the PI3K/Akt/mTOR pathway plays an important role.^193^ Overall, the entry of SARS‐CoV‐2 into host cells overactivates PI3K, which activates mTOR1 through phosphorylation of Akt. This pathway ultimately increases viral protein synthesis, inflammatory cytokine production, and affects infected cell survival. Therefore, the PI3K/Akt/mTOR pathway is an important target for COVID‐19 intervention.

### 3.7 Growth factor pathway

#### 3.7.1 TGF‐β signaling pathway

There are three isoforms of transforming growth factor‐β (TGF‐β, TGF‐β1, β2, and β3) and they are highly conserved and function similarly. TGF‐β is usually isolated as an inactive protein.^194^ It forms small noncovalent latent complex (SLC) with latency‐associated peptide. SLC is covalently bound to potential TGF‐β binding proteins (LTBP) via disulfide bonds to form large latent complex that are stored in the extracellular matrix.

During SARS‐CoV‐2 infection, expression of TGF‐β is elevated, and it is an important factor mediating ARDS, inflammation, and pulmonary fibrosis.^195^, ^196^, ^197^, ^198^ Study shows that SARS‐CoV‐2 triggers a chronic immune response directed by TGF‐β in severe COVID‐19.^199^ Growth differentiation factor 15 (GDF‐15) is a TGF‐β superfamily cytokine that responds to mitochondrial stress, tissue damage, and hypoxia, and is a strong predictor of disease severity in inflammation, cancer, and infection. In the context of COVID‐19, GDF‐15 has recently been proposed as a marker of disease severity and as a mediator of inflammation‐induced disease tolerance.^200^ Recent studies have also demonstrated that TGF‐β downregulates T‐bet leading to NK cell dysfunction in severe patients with COVID‐19.^201^

Three possible pathological relationships between SARS‐CoV‐2 and TGF‐β have been proposed: first, viral infection activates TGF‐β secretion by immune cells; second, downregulation of ACE2 receptors by spike protein and elevated levels of Ang‐II enhance intracellular SMAD2, SMAD4, and promote TGF‐β/SMAD signaling; and third, viral N protein interacts with SMAD3 to promote TGF‐β signaling activation (reviewed in Refs. 202 and 203). In addition, the article suggests possible mechanisms of TGF‐β action in SARS‐CoV‐2 infection, including reactive oxygen species (ROS)‐dependent pathways and non‐ROS pathways. Signaling pathways of the ROS‐dependent pathway include internalization of the skin sodium channel (ENaC) mediated through TGF‐β and inhibition of the antioxidant system; non‐ROS pathways include downregulation of the cystic fibrosis transmembrane conductance regulator, activation of the fibrinogen activator inhibitor 1 (PAI‐1), and NF‐kB pathway (reviewed in Ref. 203). These TGF‐β‐mediated modulations lead to inflammation and lung injury in COVID‐19 and impaired coagulation. Therefore, targeting TGF‐β and its signaling is a potential interventional therapy for COVID‐19.

#### 3.7.2 Role of VEGF in COVID‐19

VEGF has five main members: VEGF‐A, VEGF‐B, VEGF‐C, VEGF‐D, and placental growth factor. The VEGF‐VEGFR system acts to regulate angiogenesis, lymphangiogenesis, and is associated with inflammatory responses, endothelial dysfunction, and the pathogenesis of COVID‐19 (ALI, ARDS, CS, and thrombotic storms).^204^, ^205^, ^206^ In addition, VEGF may also trigger COVID‐19‐related brain inflammation by promoting the recruitment of inflammatory cells and regulating Ang II and play an important role in the development of CS and brain barrier damage.^207^ Apart from ACE, neurofibrillary protein‐1 receptor (NRP‐1 R) can act as a coreceptor through VEGF‐A/NRP‐1 involved in cell entry of SARS‐CoV‐2 spike protein.^208^ In conclusion, VEGF is overexpressed in COVID‐19, correlates with disease severity, contributes to the inflammatory process, and is involved in CS. Antagonizing VEGF may be a promising therapeutic strategy for COVID‐19.

### 3.8 S1P/S1P receptor signaling pathway

Sphingosine 1‐phosphate (S1P) is phosphorylated from the backbone sphingosine of sphingolipids and is a key signaling lipid regulator for a variety of physiological and pathological conditions, including viral infection, inflammation, immune response, vascular integrity, and CS.^209^, ^210^, ^211^ S1P was considered as a new predictor of clinical severity associated with COVID‐19.^212^ S1P/S1PR pathway is also closely associated with neuroinflammation, vascular disease, and other complications of COVID‐19.^213^, ^214^ S1P can act as an intracellular second messenger and receptor ligand, and regulation of innate and adaptive immunity is mediated primarily through binding to specific GTP‐binding protein‐coupled receptors (S1PR1‐5).^209^, ^215^ These S1PRs determine multiple responses and are discrepantly expressed in different tissues. S1P signaling responds to a variety of stimuli, including proinflammatory cytokines (TNF‐α). Activated sphingosine kinases SphK1 and SphK2 catalyze the metabolic precursors of S1P (ceramide and sphingosine) to form S1P. S1P activates its own receptors (S1PR1–5) in an autocrine or paracrine manner via transporter proteins (Spns 2, Mfsd2b, and ABCA1) to further promote S1P formation and regulate various cellular functions.^216^, ^217^, ^218^ The physiological functions of enveloped RNA viruses, such as SARS‐CoV, are dependent on host lipid biosynthesis. Much evidence suggests that the SphK/S1P/S1PR signaling pathway plays an important role in inflammatory viral infections and may be dual in nature.^210^, ^219^, ^220^, ^221^ Influenza virus infection increases intracellular SphK1/S1P signaling and activates other SphK‐mediated inflammatory signaling pathways (e.g., NF‐kB, MAPK, and PI3K/AKT) to promote viral replication, inflammatory responses, and CS.^222^, ^223^ Both SphK1 and SphK2 have a proviral role in influenza A virus (IVA) infection, and transient inhibition of targeted sphingosine kinases (SphK1 or SphK2) provides protection in mice infected with IVA.^220^ One study used SphK inhibitors and ceramidase inhibitors to block the S1P pathway, which is a possible mechanism for antimeasles virus (MV, enveloped RNA virus).^224^ However, increasing S1P levels and S1P/S1PR signaling may have therapeutic benefits for viral infection‐mediated lung injury and hyperinflammation. Influenza (H1N1) infection leads to endothelial S1PR1 deficiency, which exacerbates immune‐mediated acute lung injury, and S1PR 1 agonists play a protective function and mitigate CS and immunopathological damage during influenza virus treatment.^225^, ^226^, ^227^ Agonists of SphK, S1P, and S1PR, S1P analogs may provide effective antiviral effects.^225^, ^228^ Therefore, targeted modulation of SphK/S1P/S1PR is a promising interventional therapeutic pathway for tissue inflammation and pathological injury of COVID‐19.

## 4 INTERVENTION THERAPY

### 4.1 Antiviral and anti‐inflammatory drugs in COVID‐19

During the COVID‐19 epidemic, several antiviral and anti‐inflammatory drugs have been activated so far as potential drug candidates due to the novelty of the SARS‐CoV‐2 virus and the suddenness of the outbreak (reviewed in Refs. 229 and 230). The main mechanism of antiviral drugs is inhibition of viral replication and entry. The candidates include nucleic acid analogues (raltegravir, ribavirin, and famipiravir); protease inhibitors (lopinavir and ritonavir); and monoclonal antibodies to S proteins (sotrovimab, tixagevimab, and cilgavimab). Several broad‐spectrum immunomodulatory drugs exert anti‐inflammatory effects, including corticosteroids (dexamethasone and budesonide), statins, nonsteroidal anti‐inflammatory drugs (NSAIDs), colchicine, chloroquine/hydroxychloroquine (CQ/HCQ), N‐acetylcysteine (NAC) therapy, and intravenous immunoglobulin therapy.

Traditional Chinese medicine (TCM) has shown promising results in COVID‐19, including Qing lung detoxification soup, Lian Hua Qing Fei capsule, and other TCM formulas with antiviral and anti‐inflammatory effects (reviewed in Ref. 231 and 232). A report evaluated 578 herbal medicines and 338 reported anti‐COVID‐19 TCM formulations using high‐throughput screening combined with a bioinformatics approach.^233^ The report reveals the key targets and potential active ingredients of these herbs in relevant pathways, providing an important scientific basis for the mechanism of action of TCM in the treatment of COVID‐19 and CS.

**FIGURE 5:**
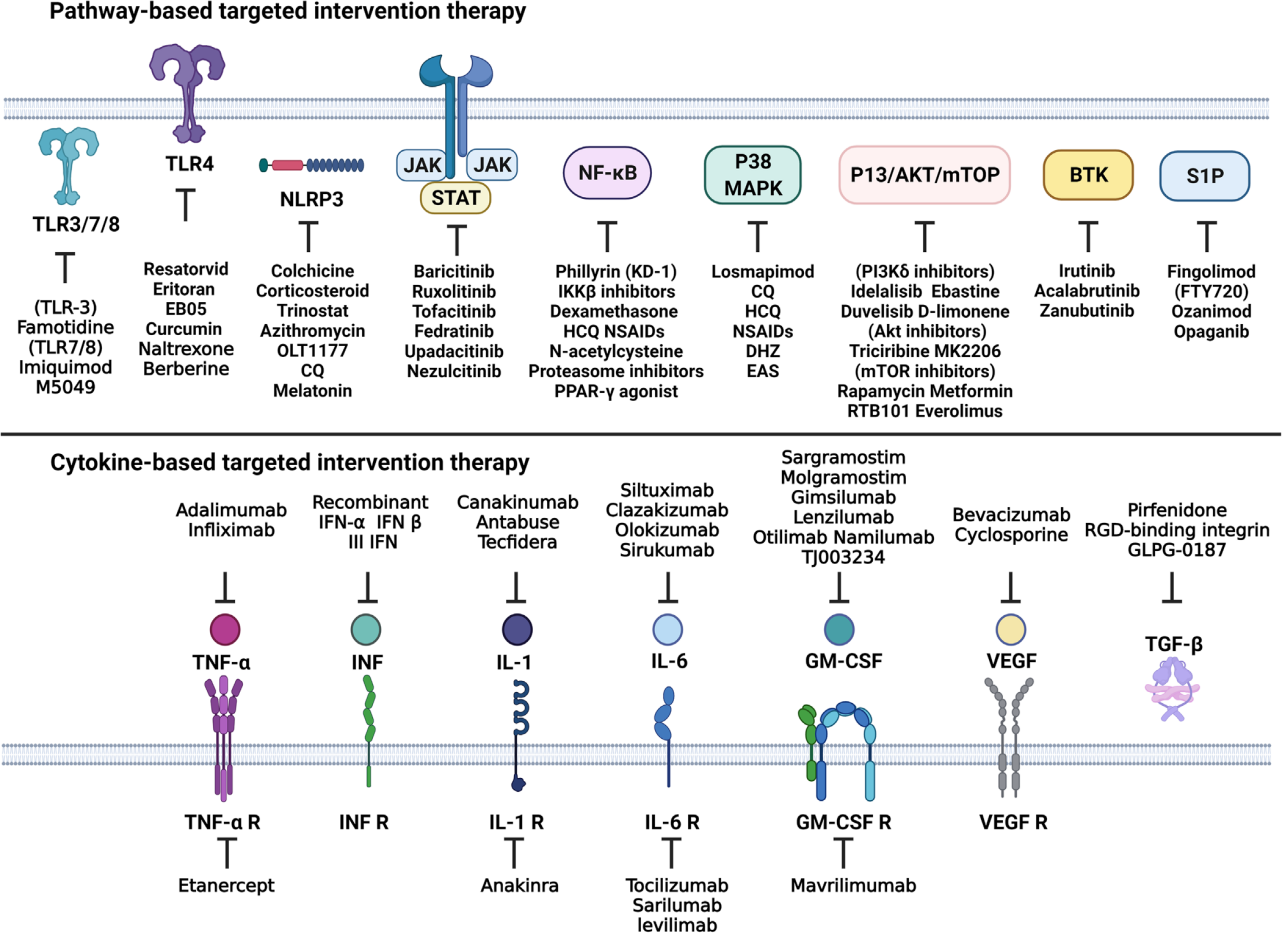
Potential therapeutic drugs targeting COVID‐19. Multiple inhibitors or drugs that have been used or are being considered clinically for the treatment of COVID‐19 include interventional therapies that target individual proinflammatory cytokines or their receptors and associated inflammatory pathways. The figure is drawn by BioRender software. Abbreviations: colchicine, CQ/HCQ, chloroquine/hydroxychloroquine; DHZ, hydrogen ginger oleone; EAS, extraction of *Asparagus officinalis* stem; NSAIDs, nonsteroidal anti‐inflammatory drugs

In addition, COVID‐19 infection leads to an ecological dysbiosis of the gut–lung axis microbiota, producing bacterial ligands, inflammatory factors, and metabolites that affect the lung flora and cause inflammation and damage to organs, such as the lung.^52^, ^234^ Therefore, we hypothesize that SARS‐CoV‐2 may affect the gut flora associated with the enteropulmonary axis to compromise human immunity and could be used to prevent and treat viral lung infections and severe COVID‐19 by modulating the associated gut flora. It was found that patients infected with SARS‐CoV‐2 had significantly lower intestinal bacterial diversity and reduced relative abundance of beneficial microorganisms, such as *Bifidobacteria*, suggesting the use of probiotics (*Bifidobacteria*, *Lactobacillus*, and *Panibacillus*) and prebiotics (foods containing prebiotics, such as fiber, oligosaccharides, and polyphenols) to effectively modulate the intestinal microbiota and prevent or mitigate COVID‐19 through the gut–lung axis (reviewed in Refs. 235, 236, 237). However, this review will focus on summarizing the targeted drugs in the COVID‐19 inflammatory pathways, focusing on the key targets and clinical implications, and these targeted interventions may provide important reference value for COVID‐19 treatment (Figure 5 and Table 1).

**TABLE 1:** Selected clinical trials and drug candidates for interventional therapy of COVID‐19 inflammatory pathways/mediators

Drug class		Drug name	Mechanism of action	Clinical trial identifier or reference
Based on pathway	TLR pathway	Resatorvid	TLR4 antagonist	^241^, ^242^
		EB05	TLR4 antagonist	NCT04479202
		Curcumin	TLR4 antagonist	NCT04382040
		Naltrexone	TLR4 antagonist	NCT04604704, NCT04604678
		Eritoran	TLR4 antagonist	NCT02735707
		Berberine	TLR4 antagonist	NCT04479202
		Famotidine	Inhibition of TLR3	NCT04389567, NCT04504240, NCT04724720, NCT04370262
		PUL‐042 inhalation solution	Block TLR2/6/9	NCT04312997, NCT04313023
		M5049	Inhibition of TLR7/8	NCT04448756
	NLRP3	Azithromycin	An anti‐inflammatory drug that blocks NLRP3	NCT04329832, NCT04332107
		Trinostat	NLRP3 inhibitor	ChiCTR2000030002, IRCT20200419047128N1
		OLT1177	Selective NLRP3 inhibitor	NCT04540120
		Melatonin	Inhibits NLRP3 activity and IL‐1 production	NCT04474483, NCT04784754, NCT04409522, NCT05318144, NCT04568863, NCT04530539, NCT04530539, NCT04570254
	JAK/STAT pathway	Baricitinib	JAK1/2 inhibitor NAK inhibitor	NCT04340232, NCT04320277, NCT04399798, NCT04421027, NCT04640168, NCT04321993, NCT04393051, NCT04373044
		Ruxolitinib	JAK1/2 inhibitor	NCT04348071, NCT04477993, NCT04414098, NCT04359290, NCT04377620, NCT04348695, NCT04374149, NCT04424056, NCT04338958, NCT04403243, NCT04334044
		Tofacitinib	JAK1/2/3, TYK2 inhibitor	NCT04332042, NCT04415151, NCT04390061, NCT04469114, NCT04412252
		Pacritinib	JAK2 inhibitor	NCT04404361
		Upadacitinib	JAK1/3 inhibitor	NCT04393311
		Nezulcitinib	pan‐JAK inhibitor	NCT05091723
	NF‐κB pathway	Phillyrin (KD‐1)	Inhibiting NF‐κB p65 and increasing IκBα	^280^
		PS‐1145, SAR113945, IKK‐16, SC‐514, BAY11‐7082, BAY11‐7085, TBK‐1, BoNT, vincristine, and resveratrol	IKKβ inhibitors	^282^
		Dexamethasone	Broad‐spectrum anti‐inflammatory or antiviral drugs that inhibit the NF‐κB pathway	NCT04325061, NCT04325061, NCT04603729, NCT05293210
		Hdroxychloroquine		NCT04340544, NCT04333225, NCT04333225, NCT04372017
		N‐acetylcysteine		NCT04545008, NCT04792021, NCT04374461, NCT04928495
		Thiazolidinedione Pioglitazone	PPAR‐γ agonists promote NF‐κB inactivation	^283^
	BTK pathway	Ibrutinib	BTK inhibitor	NCT04665115, NCT04375397, NCT04439006
		Acalabrutinib	BTK inhibitor	NCT04647669, NCT04564040, NCT04497948, NCT04380688, NCT04346199
		Zanubutinib	BTK inhibitor	NCT04382586
	P38 MAPK pathway	Losmapimod	Inhibition of p38α and p38β	NCT04511819
	P13/Akt /mTOR pathway	Duvelisib	Inhibition of PI3K‐δ and PI3K‐γ	NCT04372602
		Triciribine MK2206	Akt inhibitors	^298^
		Rapamycin	mTOR inhibitor	NCT04482712, NCT04371640, NCT04374903, NCT04341675, NCT04374903, NCT04461340, NCT04374903
		Metformin	Indirectly inhibit mTOR	NCT04604678, NCT04625985, NCT04626089, NCT04510194
		RTB101	mTOR inhibitor	NCT04584710
	S1P signaling pathway	Fingolimod (FTY720)	S1PRs (1, 3, 4, 5) agonist	NCT04280588
		Ozanimod	S1PRs (1, 5) agonist	NCT04405102
		Opaganib	SphK2 inhibitor	NCT04467840, NCT04414618, NCT04435106, NCT04502069
Based on inflammatory cytokines	IL‐6	Tocilizumab (TCZ)	IL‐6R inhibitor	NCT04306705, NCT04377659, NCT04363736, NCT04317092, NCT04403685, NCT04346355, NCT04779047, NCT05002517, NCT04479358, NCT04331808, NCT04345445, NCT04730323, NCT04380818, NCT04690920
		Sarilumab	IL‐6R inhibitor	NCT04324073, NCT04386239, NCT04357808, NCT04322773, NCT04357860, NCT04661527, NCT04315298,NCT02735707, NCT04324073, NCT04359901
		Siltuximab	L‐6 inhibitor	NCT04329650, NCT04330638, NCT04486521
		Sirukumab	L‐6 inhibitor	NCT04380961
		Clazakizumab	L‐6 inhibitor	NCT04381052, NCT04494724, NCT04363502, NCT04659772, NCT04343989
		Levilimab	IL‐6R inhibitor	NCT04397562
		Olokizumab	L‐6 inhibitor	NCT04380519, NCT04452474
	IL‐1	Canakinumab	IL‐1 β antagonist	CT04476706, NCT04348448, NCT04362813, NCT04365153, NCT04510493, NCT04278404
		Anakinra	IL‐1R antagonist	^355^
		Disulfiram Dimethyl fumarate (Tecfidera)	GSDMD inhibitors block IL‐1β release	NCT04485130, NCT04594343
				NCT04381936
	TNF‐α	Adalimumab	TNF‐α inhibitor	NCT04705844, ChiCTR2000030089
		Infliximab	TNF‐α inhibitor	NCT04922827, NCT04734678, NCT05220280, NCT04593940, NCT04425538
	Interferon (IFN)	Recombinant IFN‐α	Immunomodulation Viral clearance	NCT04480138, NCT04988217, NCT04379518, NCT04320238, NCT04293887
		Recombinant IFN‐β		NCT04449380, NCT04343768, NCT04492475, NCT04350281, NCT04324463, NCT04647695, NCT04494399
	GM‐CSF	Sargramostim	Human recombinant GM‐CSF	NCT04411680, NCT04326920, NCT04707664, NCT04642950, NCT04400929
		Molgramostim	Human recombinant GM‐CSF	NCT04569877
		Mavrilimumab	GM‐CSF receptor inhibitor	NCT04447469, NCT04492514 NCT04463004, NCT0439998, NCT04397497
		Gimsilumab	GM‐CSF inhibitor	NCT04351243
		Otilimab	GM‐CSF inhibitor	NCT04376684
		Lenzilumab	GM‐CSF inhibitor	NCT04351152
		TJ003234	GM‐CSF inhibitor	NCT04341116
Based on growth factors	TGF‐β	Pirfenidone	An anti‐inflammatory drug that downregulates TGF‐β gene expression	NCT04653831, NCT04607928, NCT04856111
	VEGF	Bevacizumab	VEGF inhibitor	NCT04305106, NCT04344782, NCT04954014, NCT04275414, NCT04822818
		Cyclosporine	An immunosuppressant that downregulates T cell and VEGF activity	NCT04492891, NCT04979884, NCT04451239, NCT04492891, NCT04979884, NCT04451239

*Note*: ChiCTR2000030089 and ChiCTR2000030002 are from the China Clinical Trials Registry (www.chictr.org.cn). IRCT20200419047128N1 is from the Iranian Clinical Trials Registry (www.irct.ir). Other clinical trial information from ClinicalTrials.gov.

Abbreviations: Akt, protein kinase B; BTK, Bruton's tyrosine kinase; GM‐CSF, granulocyte‐macrophage colony‐stimulating factor; JAK, janus kinase; MAPK, mitogen‐activated protein kinase; mToR, mammalian target of rapamycin; NF‐κB, nuclear factor kappa‐B; NLRP3, nod‐like receptor family, pyrin domain‐containing 3; PI3K, phosphatidylinositol 3‐kinase; S1P, sphingosine 1‐phosphate; TGF‐β, tumor growth factor‐β; TLR, toll‐like receptor; TNF‐α, tumor necrosis factor‐α; VEGF, vascular endothelial growth factor.

### 4.2 Interventional therapy based on sensory receptors

#### 4.2.1 TLR interventions

A number of studies have explored the critical role of TLR in COVID‐19, both in mediating host protection mechanisms of innate immunity and in inducing CS, which cause excessive inflammation and tissue damage in COVID‐19 immunopathology.^64^, ^65^, ^238^ Therefore, interventions targeting the TLR pathway should consider controlling TLR activity to restore immune homeostasis in COVID‐19 patients. Currently, targeting TLR as a potential immunotherapeutic strategy for COVID‐19 mainly includes TLR agonist/antagonist and as an adjuvant or target of anti‐SARS‐CoV‐2 vaccine (reviewed in Refs. 238, 239, 240).

One study reported that the spike protein of SARS‐CoV‐2 has the strongest binding affinity to TLR4.^67^ TLR4 antagonists are an effective strategy for the treatment of COVID‐19. Resatorvid (called CLI‐095 or TAK‐242) is a TLR inhibitor that selectively binds TLR4 and blocks its interaction with the junctional proteins (TIRAP and TRAM), thereby inhibiting TLR4/MyD88/NF‐κB signaling and NLRP3 activation.^241^, ^242^ TLR4 modulators, such as Eritoran (NCT02735707), EB05 (NCT04401475), curcumin (NCT04382040), naltrexone (NCT04604704 and NCT04604678), and berberine (NCT04479202), are in clinical trials for COVID‐19. In addition, HMGB1/TLR4, a therapeutic target for severe pneumonia, including COVID‐19, mediates the release of proinflammatory cytokines, and acetylcholine, heparin, statins, glycopyrrolate, ketamine, and resveratrol are important drug candidates as TLR4 inhibitors.^243^ Famotidine reduces TLR3‐dependent signaling and ultimately reduces IRF3 and NF‐κB pathway‐induced inflammation and cytokine release.^244^ Studies have shown that prophylactic administration of TLR2/6 agonists can reduce the transmission of SARS‐CoV‐2 and provide protection to some extent.^245^ Imiquimod, a natural immune agonist of TLR7/8, induces the production of proinflammatory cytokines and is suggested to play an antiviral role as early COVID‐19 treatment.^246^ Activation of TLR7/8 may have a dual role in COVID‐19 disease progression, and a clinical trial in China (ChiCTR2000029776) evaluated the effect of induction of TLR pathways for the treatment of COVID‐19. In addition, a clinical study of a novel TLR7/8 inhibitor (M5049, NCT04448756) has been completed with unpublished results.

During the design and development of vaccines against SARS‐CoV‐2, some vaccines used TLR agonists as adjuvants in the vaccine design to enhance the efficacy of COVID‐19 vaccine; some vaccines were found to act on TLR (TLR3/4/5) and regulate downstream signaling through molecular docking (reviewed in Refs. 239 and 240). Such vaccines targeting TLRs could be used for immunotherapeutic intervention in SARS‐CoV‐2 infection.

#### 4.2.2 NLRP3 inhibitors

Various studies have shown that NLRP3 is an important target for COVID‐19 intervention.^247^, ^248^ Blocking NLRP3 provides a valuable therapeutic option for COVID‐19. Colchicine is an anti‐inflammatory and immunomodulatory drug that inhibits NLRP3 by inhibiting the P2×7 receptor and caspase‐1 activation.^249^ Thirty‐seven clinical studies on colchicine are currently registered on clinicaltrials.gov and have reported no effect on clinical events and mortality, but a beneficial effect on the reduction of hospital stays and adverse events.^250^, ^251^ Therefore, it can be a useful and inexpensive option for the treatment of COVID‐19. In addition, anti‐inflammatory drugs that target caspase‐1 to block NLRP3 include corticosteroid drugs (dexamethasone and methylprednisolone) and SERM raloxifene.^252^ Early IFN therapy may also inhibit the NLRP3 inflammasome, providing a benefit to COVID‐19 (see Section 4.4.4 for details). Several selective or nonselective anti‐inflammatory agents can block NLRP3 and its signaling, such as trinostat, azithromycin, dapansutrile (OLT1177), and chloroquine (reviewed in Refs. 78 and 247). Many clinical studies are underway with azithromycin (e.g., NCT04329832 and NCT04332107) against COVID‐19 and it is often used together with chloroquine. Trinostat has conducted two clinical studies for COVID‐19 in Iran (IRCT20200419047128N1) and China (ChiCTR2000030002). One clinical study on OLT1177 (NCT04540120) is being recruited to evaluate its safety and efficacy.

In recent years, NLRP3 has been identified as a new target for melatonin action, and melatonin inhibits NLRP3 activity and IL‐1 production through multiple pathways.^253^ Eleven clinical studies, including safety, efficacy, and dosing (e.g., NCT04784754, NCT04474483, and NCT04353128), have been conducted with melatonin in COVID‐19. The use of cytokine inhibitors, such as IL‐1 inhibitors, is also a way to block the NLRP3/IL‐1 pathway (see Section 4.4.2 for details). In addition, studies have shown that some natural drugs, including curcumin, chamomile lactone, Bay11‐7082, oridonin, and flavonoids, exert anti‐inflammatory functions by inhibiting NLRP3.^254^, ^255^, ^256^

### 4.3 Signaling pathway intervention therapy

#### 4.3.1 JAK/STAT signaling pathway and JAK inhibitors

JAK/STAT is a signaling pathway for multiple inflammatory cytokines in COVID‐19, and its properties in inflammation and immune regulation can be used to manage CS, as many studies have used JAK/STAT signaling inhibition as an intervention therapy for targeting COVID‐19 (reviewed in Refs. 87, 88, 125, 257, 258, 259). Small molecule JAK inhibitors (JAKinibs), biologics that bind to and inhibit JAK‐catalyzed ATP sites, have been used to treat many inflammation‐driven diseases, such as inflammatory bowel disease, RA, and psoriatic arthritis, as well as emergency authorizations for COVID‐19 therapy.^260^ The main JAK inhibitors used for COVID‐19 interventions in current clinical studies include baricitinib, ruxolitinib, and tofacitinib.

Baricitinib is a highly selective kinase inhibitor that effectively and reversibly inhibits JAK1 and JAK2, and inhibits proinflammatory signaling by a variety of cytokines, including IL‐6, IL‐1β, IL‐12, GM‐CSF, and IL‐2.^261^, ^262^ A recent preliminary clinical study has shown that baricitinib has a good safety and efficacy profile for intervention in COVID‐19 patients without side effects.^263^ In addition, baricitinib blocks viral endocytosis and assembly by inhibiting AP2‐associated protein kinase (AAK1) and the cell cycle protein G protein‐associated kinase.^125^, ^261^ Baricitinib also improves lung function in patients with moderate/severe COVID‐19 treated with corticosteroids and suppresses immune dysregulation in patients with severe disease.^264^, ^265^ Some other reports have also published results.^264^, ^266^, ^267^, ^268^, ^269^, ^270^, ^271^ Baricitinib is clearly a safer treatment for patients with moderate/severe COVID‐19. However, as an immunosuppressant, baricitinib may increase the risk of infection in patients with severe disease. Prospective studies on it (NCT04320277 and NCT04321993) are ongoing.

Ruxolitinib is a potent JAK1/2 inhibitor that also has moderate effects on TYK2 and JAK3, blocking STAT activation and nuclear translocation by terminating JAK kinase activity. It significantly reduces cytokine levels, improves hyperinflammation, reduces lung injury, and rapidly restores lymphocyte counts by inhibiting the JAK/STAT signaling pathway.^88^, ^272^ Several clinical studies have reported that ruxolitinib effectively treats severe/critical COVID‐19 patients associated with ARDS by inhibiting cytokine signaling(reviewed in Ref. 272). It was also demonstrated to be safe and effective in a multicenter phase II clinical trial (NCT04348071) in COVID‐19 interventions characterized by CSS. Among the current interventions for COVID‐19, ruxolitinib may be an effective treatment for severe/critical COVID‐19 and has shown good tolerability in elderly patients.

Tofacitinib is a JAK1/2/3 inhibitor approved for RA that primarily inhibits JAK1 and JAK 3 and, to a lesser extent, JAK2 and TYK 2.^273^ Tofacitinib is currently less studied in clinical studies of COVID‐19 than ruxolitinib and baricitinib JAK inhibitors. Pfizer designed a multicenter study, randomized and double‐blind, a placebo‐controlled, 260‐participant clinical trial of tofacitinib (NCT04469114) to study its safety and efficacy in patients with COVID‐19 hospitalized for pneumonia and receiving standard of care treatment. A clinical study (NCT04415151), also conducted at Yale University, looks at the therapeutic potential of tofacitinib in patients with moderate COVID‐19.

There are other JAK inhibitors that may be candidates for COVID‐19 intervention therapy. Meolitinib and oclacitinib are JAK1/2 inhibitors that block the downstream signaling pathways of most proinflammatory factors. Fedratinib is a highly selective JAK2 inhibitor that has been approved for myelofibrosis treatment.^274^ Studies have reported the involvement of Th17‐type CRS in the progression of severe immune damage in COVID‐19.^275^ Fedratinib inhibits IL‐17 expression in Th17 cells and could be considered for its potential role in COVID‐19 treatment. Pacritinib, a JAK2 inhibitor, has been studied in prevention studies in critical COVID‐19 patients with or without cancer (NCT04404361). Upadacitinib, a selective JAK1/3 inhibitor for the treatment of severe RA, inhibits IL‐6, GM‐CSF, and IFN‐γ production and has anti‐inflammatory potential for COVID‐19, and currently has one clinical trial registration (NCT04393311).^276^, ^277^ Nezulcitinib is an inhaled pan‐JAK inhibitor that has recently been reported to have good therapeutic potential in respiratory tract injury and COVID‐19.^278^, ^279^ Currently, there is one clinical study (NCT05091723) registered on its treatment of COVID‐19 acute lung injury.

#### 4.3.2 NF‐κB signaling pathway and inhibitors

The NF‐κB pathway plays a central role as an immune switch in COVID‐19‐induced hyperinflammation and CS. Inhibition of the NF‐κB pathway would inhibit the release of multiple proinflammatory cytokines, chemokines, and adhesion molecules, and is an important potential therapeutic target for COVID‐19. Phillyrin (KD‐1) is the active component of forsythia with anti‐inflammatory, antioxidant, and antiviral properties. An in vitro study showed that KD‐1 inhibits NF‐κB signaling by decreasing the activation of NF‐κB p65 and increasing the expression of IκBα, which is a novel strategy to control COVID‐19.^280^ Another in vitro study identified a novel pyrazole analogue (C6) as an inhibitor of NF‐ĸB transcriptional activity and a positive regulator of IĸBα.^281^ It significantly inhibits various proinflammatory cytokines and is a potential precursor for treating the COVID‐19 inflammatory response. It has been summarized that pharmacological inhibitors targeting IKKβ (a major downstream effector of the NF‐κB pathway), such as PS‐1145, SAR113945, IKK‐16, BAY11‐7082, BAY11‐7085, SC‐514, TBK‐1, BoNT, vincristine, and resveratrol, can be potential strategies against severe COVID‐19.^282^ TNFα is a key proinflammatory factor for NF‐κB pathway activation, and inhibitors targeting TNF‐α may also reduce NF‐κB signaling (see Section 4.4.3 for details). Several commonly used broad‐spectrum anti‐inflammatory or antiviral drugs are reused in COVID‐19, such as dexamethasone, HCQ, macrolide antibiotics, NSAIDs (e.g., acetylsalicylic acid and aspirin), remdesivir, NAC, and proteasome inhibitors (VL‐01, bortezomib, carfilzomib, and lxazomib) are associated with NF‐κB cascade inhibition (reviewed in Refs. 135, 139, and 140). Peroxisome proliferator‐activated receptor (PPAR)‐γ, a member of the PPAR transcription factor family, regulates inflammation mainly by promoting NF‐κB inactivation in different ways, including silencing proinflammatory gene inducers of NF‐κB, interacting with p65, and reducing ROS production.^283^ Therefore, synthetic agonists of PPAR‐γ (thiazolidinedione and pioglitazone) and its natural ligands (turmeric, seafood, and lemongrass) can be used as references for the regulation of COVID‐19.

In addition, the NF‐κB pathway usually crosstalks with multiple pathways to exert a joint anti‐inflammatory effect, therefore, interventional drugs of other pathways (JAK inhibitors, IL‐6 inhibitors, BTKis, and MAPK inhibitors) will also play a beneficial role in inhibiting the NF‐κB pathway.

#### 4.3.3 BTK inhibitors and COVID‐19

BTK has a complex role in COVID‐19, which regulates both B cell and macrophage signaling, development, and activation, and also interacts with multiple inflammatory pathways (PI3K, NF‐κB, MAPK, and NLRP3) (reviewed in Refs. 163 and 284). Therefore, BTKis are beneficial for inflammatory responses, lung injury, thrombosis, and tumors in COVID‐19 patients.^164^ Clinically, the BTKis used for COVID‐19 are ibrutinib, acalabrutinib, and zanubutinib. Ibrutinib is a first‐generation BTKi that selectively and irreversibly inhibits BTK regulation of B cells and cytokine release. In a clinical report, six WM patients infected by COVID‐19 were treated with ibrutinib and the results showed that ibrutinib prevented lung injury from COVID‐19.^285^ There are currently three ongoing clinical trials on ibrutinib (NCT04665115, NCT04375397, and NCT04439006) and evaluating its effectiveness in COVID‐19 patients. An observational study used acalabrutinib to treat 19 severe COVID‐19 patients who required supplemental oxygen, and its results showed that acalabrutinib contributed to improved oxygenation, inflammation, and prognostic clinical status of the patients.^286^ Currently, three clinical trials (NCT04497948, NCT04380688, and NCT04346199) evaluating acalabrutinib for optimal treatment have been completed, as well as two additional ongoing trials (NCT04647669 and NCT04564040). Zanubrutinib is a second‐generation BTKi with lower toxicity than the first generation, and one clinical study (NCT04382586) has been completed to evaluate its therapeutic potential in COVID‐19, whose results have not reach the endpoint. In conclusion, BTKi may play a beneficial role in the management of COVID‐19, but we still need well‐designed clinical studies to assess its effectiveness.

#### 4.3.4 MAPK signaling pathway inhibitors

Mechanisms of MAPK pathway dysregulation promote SARS‐CoV‐2 infection and inflammatory injury, and in particular, the P38 MAPK pathway is an important participant in the pathogenesis of COVID‐19. Therefore, targeting MAPK in COVID‐19 is a feasible intervention or treatment. Quite a few inhibitors targeting P38 MAPK have entered clinical trials, among which losmapimod is the most clinically studied p38 inhibitor with a good safety profile.^166^ It works by inhibiting p38α and p38β, which, in turn, blocks downstream signaling. A clinical trial evaluating the efficacy of losmapimod (NCT04511819) has been conducted in COVID‐19 patients. Although the trial was terminated due to changes in the treatment setting and participant recruitment, p38 inhibitors remain promising candidates for COVID‐19. Studies have highlighted that CQ, HCQ, and their derivatives are broad‐spectrum antiviral agents that have potential as MAPK cascade inhibitors in COVID‐19.^287^ CQ or NSAIDs can also upregulate DUSP1 and DUSP5 gene expression to indirectly inhibit MAPK/NF‐κB signaling.^179^ In addition, some natural compounds exert anti‐inflammatory effects by inhibiting MAPK through different mechanisms. Hydrogen ginger oleone (DHZ) regulates MAPK and NF‐κB pathways to inhibit CS, oxidation, and provide preclinical support for ARDS.^288^ Extraction of *Asparagus officinalis* stem inhibits p44/42 MAPK and Akt signaling in macrophages and helps to alleviate excessive inflammation.^289^

#### 4.3.5 P13/Akt/mTOR signaling pathway inhibitors

This paper reviews the emerging role of the PI3K/Akt/mTOR pathway in COVID‐19 infection. Single or combined inhibitors targeting PI3K, Akt, and mTOR may have potential therapeutic effects in the course of SARS‐COV‐2 infection.^290^, ^291^ Among them, the most widely studied are mTOR inhibitors.^292^

##### 4.3.5.1 P13 inhibitors

Much prior evidence suggests that the PI3K pathway affects T cell, B cell survival, memory cell differentiation, and inflammatory factor production, which is an anti‐inflammatory target in airway diseases (asthma and allergic rhinitis).^293^, ^294^ In particular, PI3Kδ isoform inhibitors are considered as a valuable approach for the treatment of inflammatory lung diseases.^295^, ^296^ Therefore, PI3Kδ inhibitors idelalisib and ebastine, which have the effect of reducing inflammatory cytokines and proinflammatory cell (T cell) activation, migration, have been proposed as candidates for COVID‐19 interventional therapy.^296^ Duvelisib inhibits both PI3K‐δ and PI3K‐γ, and a clinical study on its treatment of COVID‐19 clinical study (NCT04372602) is ongoing. In addition, D‐limonene is a monoterpene whose pharmacological mechanism is to inhibit PI3K binding to NF‐κB p65 and block its downstream signaling pathway.^297^ D‐limonene has potential therapeutic value against COVID‐19‐mediated inflammation and pulmonary fibrosis.

##### 4.3.5.2 Akt inhibitors

Targeted inhibition of Akt, which may downregulate ACE2 expression in COVID‐19 and may increase Tregs, inhibits excessive inflammation, CS, pulmonary fibrosis, and platelet activation associated with advanced COVID‐19.^298^ Akt inhibitors are emerging as potential drug options, such as triciribine and MK2206, which are recommended alone or in combination with the current standard of care for treating advanced COVID‐19 and ARDS.^298^

##### 4.3.5.3 mTOR inhibitors

The mTOR 1 is polymorphic in the immune response to viral infection.^299^, ^300^ It promotes Th1, Th2, and Th17 cell differentiation, B‐cell development, and reduces Treg differentiation, reduction of mTOR interferes with CD8^+^ T cell proliferation. The mTOR cascade responses are also commonly associated with hypermetabolic activity. SARS‐CoV‐2 infection can overactivate mTOR1, which ultimately leads to inflammation and immunopathological damage.^301^, ^302^ A biological assessment of immunity in COVID‐19 patients suggests that viral infection leads to spatial differences in innate and adaptive immune responses.^303^ Targeted inhibition of the mTOR cascade response is a possible tool for recovery.^303^, ^304^

Rapamycin (sirolimus), as a suitable mTORC1 inhibitor, becomes an effective interventional treatment for COVID‐19.^305^ Rapamycin specifically inhibits mTORC1 by binding to the FKBP12‐rapamycin (FRB) domain of mTORC1 and blocking its interaction with the mTOR regulatory‐associated protein (raptor).^306^ Rapamycin is also known as an antiaging drug, which controls the development of CS and COVID‐19 by inhibiting protein synthesis, viral replication, and lymphocyte activation on the one hand, and exerts antiaging and antiobesity effects on the other hand.^305^, ^307^, ^308^ Although rapamycin is considered to be a good potential therapeutic agent, there yet lacks supporting evidence from clinical trials. The studies on the efficacy of rapamycin in clinical COVID‐19 patients NCT04482712 and NCT04371640 have been withdrawn, and the clinical trial of rapamycin in combination with HCQ (NCT04374903) is difficult to move forward. In addition, a number of other mTOR inhibitors, such as metformin (targets AMPK to indirectly inhibit mTOR), everolimus (inhibits mTORC1), RTB101 (inhibits mTORC), sapanisertib (inhibits mTORC1 and mTORC2), and PP‐242 (inhibits mTORC1 and mTORC2), have also been included as candidates for COVID‐19 treatment (reviewed in Refs. 290 and 292). To date, NCT04341675 (rapamycin), NCT04461340 (rapamycin), NCT04374903 (rapamycin), and NCT04584710 (RTB101) remain active for recruitment and preparation, with the hope of seeing surprising progress in the future.

#### 4.3.6 Agonist of S1P signaling pathway

The SphK/S1P/S1PR pathway was previously discussed as an important player in viral infection and inflammation, and many data strongly support that modulating the components of SphK/S1P/S1PR provides beneficial assistance in the management of COVID‐19 disease (reviewed in Refs. 309, 310, 311). Currently, drugs that modulate SphK/S1P/S1PR are being reconsidered for COVID‐19 interventions. Fingolimod (FTY720) is a novel immunosuppressive agent that belongs to S1PR immunomodulators and can be one of the candidates for COVID‐19 treatment. FTY720 is an S1P analogue that is phosphorylated as a substrate by SphK1 and SphK2 and converted to FTY720‐P.^312^ FTY720 is an unselected agonist for four S1PRs (S1PR 1, 3, 4, and 5), which is phosphorylated to exert immunosuppressive effects.^313^ FTY720‐P binds selectively to S1PR1 and leads to downregulation and internalization of S1PR1, which acts to reduce lymphocytes and suppress inflammatory responses.^313^, ^314^ Thus, FTY720 has a potentially positive effect on suppressing excessive inflammation or CS in COVID‐19. Currently, there is only one clinical trial investigating the efficacy of FTY720 in the treatment of COVID‐19 (NCT04280588) and is in withdrawal status. Similar to FTY720, the FDA approved another more specific S1PR (S1PR 1, 5) agonist drug, ozanimod (RPC1063).^315^ Compared with FTY720, ozanimod restores lymphocytes in a shorter period of time and does not show cardiac conduction abnormalities or hypertension, which may bring a better clinical benefit.^316^ Opaganib (ABC294640) is a selective inhibitor of SphK2 that reduces intracellular levels of S1P and its signaling‐induced inflammatory pathways by competitively binding SphK2.^317^ Recently, opaganib has been classified as a therapeutic agent for COVID‐19 patients, and four clinical trials are currently underway to investigate its efficacy (NCT04467840, NCT04414618, and NCT04435106), one of which was withdrawn (NCT04502069).

### 4.4 Cytokine‐based interventional therapies

#### 4.4.1 IL‐6 inhibitors

IL‐6 is a pleiotropic cytokine with high activity in SARS‐CoV‐2 infection and cancer, and its high expression leads to excessive inflammation and CS and is associated with SARS‐CoV‐2‐induced multiorgan failure (reviewed in Ref. 318). It also stimulates the occurrence of events, such as hypoxia, hypoxemia, abnormal angiogenesis, and chronic inflammation that are interrelated with COVID‐19.^318^ Therefore, IL‐6 is used as an important cytokine drug target for the interventional treatment of COVID‐19. Currently, some of the drugs used clinically to target IL‐6 signaling mainly include IL‐6 inhibitors (siltuximab, clazakizumab, sirukumab, and olokizumab) and IL‐6R inhibitors (tocilizumab [TCZ], sarilumab, and levitimab), among which the IL‐6 blockers approved for COVID‐19 clinical studies are TCZ, sarilumab, and siltuximab (reviewed in Ref. 108).

TCZ is a humanized monoclonal antibody (mAb) that specifically binds mIL‐6R and sIL‐6R and inhibits downstream signal transduction. In a study in China, 21 patients with severe/critical COVID‐19 received intravenous TCZ (4–8 mg/kg) and demonstrated improved oxygenation, reduced CRP concentrations, decreased inflammation, rapid recovery, and minimal side effects, along with good clinical benefit and therapeutic potential.^319^ This suggests that TCZ has good clinical benefit and therapeutic potential for the treatment of severe COVID‐19. Other clinical studies on TCZ for the treatment of severe COVID‐19 and its associated hyperinflammation, ARDS, and CS have also reported similar effects.^320^, ^321^, ^322^, ^323^, ^324^, ^325^, ^326^ Several reports have shown that TCZ for the treatment of severe COVID‐19 is not only safe and effective but also reduces mortality, respiratory failure, ICU admissions, and the need for mechanical ventilation.^327^, ^328^, ^329^, ^330^, ^331^, ^332^, ^333^ Recently published conclusions from systemic evaluations of TCZ therapy suggest similar positive effects.^334^, ^335^, ^336^ However, some of the TCZ studies on COVID‐19 treatment have presented conflicting results or discrepancies in reducing mortality, ICU admissions, and mechanical ventilation.^337^, ^338^, ^339^, ^340^, ^341^ Overall, a total of 96 clinical trials of TCZ for the treatment of COVID‐19 are currently registered on ClinicalTrials.gov. Although not all studies have had equal benefits, TCZ has been proven to be a safer option for patients with severe COVID‐19 and may reduce the progression of mechanical ventilation and death.

Sarilumab is a mAb humanized against the sIL‐6 and mIL‐6 receptors. Compared to TCZ, both of which have the same mechanism of action, sarilumab is also a good choice for severe COVID‐19, but it has a higher capacity and affinity for IL‐6R, suggesting that it can be administered at lower doses.^342^ The results of three clinical trials from Italy on severe COVID‐19 showed that sarilumab has a good efficacy and safety profile, improving oxygenation, reducing inflammatory biomarkers, and promoting patient recovery.^343^, ^344^, ^345^ However, some of the clinical studies showed suboptimal results, with sarilumab showing no statistically significant difference in overall improvement and mortality in severe COVID‐19, nor showing better clinical benefit.^346^, ^347^, ^348^ There are currently 18 clinical trials registered on ClinicalTrials.gov related to the treatment of COVID‐19 with sarilumab, and for demonstrating the efficacy of sarilumab in severe COVID‐19 patients, it still demands larger and more comprehensive clinical studies.

Siltuximab is a chimeric human mAb that inhibits IL‐6 activity and its downstream signaling by blocking IL‐6 binding to the receptor. Although no studies have been reported to support the results of using siltuximab for COVID‐19, a nonpeer‐reviewed observational report from Italy suggests that siltuximab (11 mg/kg) administration was well tolerated and reduced CRP and mortality in patients.^349^ And three other clinical trials evaluating the efficacy and safety of siltuximab in severe COVID‐19 are ongoing (NCT04329650, NCT04330638, and NCT04486521).

A number of other IL‐6 blockers for the treatment of severe COVID‐19 are ongoing, including sirukumab (NCT04380961); clazakizumab (NCT04381052, NCT04494724, NCT04363502, NCT04659772, and NCT04343989); levilimab (NCT04397562); and olokizumab (NCT04380519 and NCT04452474), which may become important drug candidates in the future.

#### 4.4.2 IL‐1 inhibitors

IL‐1β is a highly proinflammatory factor that promotes excessive inflammation and CS in COVID‐19 through activation and circulation of the NF‐κB pathway, therefore, blocking IL‐1β may be an effective strategy for COVID‐19 treatment. The IL‐1β antagonist canakinumab and the IL‐1 receptor antagonist anakinra are currently used primarily as possible agents for the treatment of COVID‐19 and provide clinical benefit. Canakinumab is a human mAb that neutralizes IL‐1β. Its first retrospective analysis of treated COVID‐19 patients reported the safety and efficacy of canakinumab (300 mg), which significantly improved oxygenation and systemic inflammatory response.^350^ A prospective report of canakinumab showed that it rapidly restored normal oxygenation and was associated with a good prognosis.^351^ Similar results were reported in another Italian study in patients with mild and severe COVID‐19.^352^ These results suggest that canakinumab has good therapeutic potential for COVID‐19, and six clinical trials on canakinumab have been registered (NCT04476706, NCT04348448, NCT04362813, NCT04365153, NCT04510493, and NCT04278404).

The first report of anakinra for COVID‐19 suggests that in severe COVID‐19‐induced CS, anakinra may be a safe and promising strategy for effectively reducing inflammation and prevent multiorgan dysfunction.^353^ A retrospective report of high‐dose anakinra in patients with COVID‐19, moderate to severe ARDS, and excessive inflammation showed safe outcomes, with no recurrence of inflammation after discontinuation in the treatment group, and was associated with clinical improvement in 72% of patients.^354^ Many clinical benefits of anakinra treatment for COVID‐19 have been demonstrated, particularly in patients with excessive inflammation. A review of IL‐1β blockers for the treatment of COVID‐19 summarizes the details of a dozen case reports of anakinra for the treatment of COVID‐19 patients and the progress of canakinumab studies.^355^ The results suggest that anakinra is a safe, effective, and promising treatment strategy for COVID‐19, showing good clinical benefit especially in patients with excessive inflammation. In addition, two FDA‐approved GSDMD inhibitors, disulfiram (Antabuse) or dimethyl fumarate (Tecfidera), are key mediators in blocking focal death and IL‐1β release and are currently being used to assess clinical efficacy (NCT04485130, NCT04594343, and NCT04381936).^356^, ^357^

#### 4.4.3 TNF‐α inhibitors

TNF‐α is a highly proinflammatory cytokine, which is involved in the activation of NF‐κB pathway and its vicious cycle, thus blocking TNF‐α is a potential therapeutic strategy for hyperinflammation and CS in COVID‐19.^147^, ^358^, ^359^, ^360^ The current use of inhibitors against TNF‐α for the treatment of COVID‐19 mainly includes monoclonal antibodies, such as adalimumab, golimumab, infliximab, certulizumab, and the TNF‐α receptor fusion protein etanercept. A clinical study (NCT04425538) reported results that infliximab treatment improved oxygenation and eliminated pathological inflammatory signals.^361^ Most of these are still being evaluated for their clinical effectiveness. Adalimumab is registered in two clinical trials (NCT04705844 and ChiCTR2000030089), and infliximab is also in four clinical trials (NCT04922827, NCT04734678, NCT05220280, and NCT04593940).

#### 4.4.4 IFN therapy

SARS‐CoV‐2 infection can antagonize the IFN response through multiple mechanisms.^362^ In recent years, it has been reported that ORF3b, ORF6, ORF8, and N proteins of SARS‐CoV‐2 cause IFN dysregulation, which exhibits low levels of I IFN and III IFN in COVID‐19 patients.^119^, ^363^, ^364^ Therefore, enhancing IFN response is an effective means of antiviral therapy. The main IFNs currently approved for therapeutic use are recombinant IFN‐α (IFN α‐2a/2b, IFN α‐n1/n3, and I IFN α‐con 1) and IFN β (IFN β‐1a and IFN β‐1b).^365^ Many clinical studies are ongoing with IFN or in combination with other antivirals (lopinavir/ritonavir, ribavirin, and raltegravir) for the treatment of COVID‐19.^45^, ^366^ The current partial view is that IFN‐α reduces viral shedding and inflammatory markers, while IFN‐β is primarily associated with viral clearance.^367^ A clinical study from China using IFN‐α2b and arbidol for COVID‐19 showed that the treatment group could significantly reduce the duration of virus persistence in the upper respiratory tract and the level of proinflammatory factors (IL‐6).^368^ Results from several clinical trials on the efficacy and safety of IFN‐β for the treatment of severe COVID‐19 have shown that IFN‐β significantly reduces patient mortality and improves hospital discharge rates.^369^, ^370^ However, some other clinical data using IFN‐β and in combination with other antivirals showed no significant effect of IFN‐β treatment on in‐hospital survival, hospital discharge, and mortality.^371^, ^372^ Here are several reviews that summarize the potential and clinical significance of I IFN for the treatment of COVID‐19 in recent years.^373^, ^374^, ^375^ In conclusion, although the effectiveness of I IFN remains controversial, IFN‐I may be an effective prophylactic agent or early treatment option for COVID‐19. IFN‐α therapy may provide clinical benefit for COVID‐19, and INF‐β therapy may be a potentially effective treatment strategy for COVID‐19. In addition, III IFN has been suggested as an emerging therapeutic option for COVID‐19, although relevant clinical data are yet insufficient for supporting this.^119^

#### 4.4.5 GM‐CSF therapy

GM‐CSF, as an important immune cytokine, mediates the activation of multiple immune cells at steady state, which facilitates early host defense.^127^, ^128^ However, in the pathogenesis of COVID‐19, dysregulated GM‐CSF leads to excessive inflammation, CS, and ARDS. Considering the potentially protective role of GM‐CSF in early viral infection, two human recombinant GM‐CSF, sargramostim and molgramostim, are used to evaluate their therapeutic efficacy in COVID‐19. Six major clinical studies are currently underway, five on sargramostim (NCT04411680, NCT04326920, NCT04707664, NCT04642950, and NCT04400929), and one on molgramostim is being recruited (NCT04569877). A published result of sargramostim (NCT04411680) showed that it improved oxygenation and pulmonary compliance in patients with severe sepsis and ARDS. In severe COVID‐19 cases, inhibition of GM‐CSF signaling may be more helpful in improving excessive inflammation and lung injury. Six monoclonal antibodies against GM‐CSF (mavrilimumab, gimsilumab, lenzilumab, otilimab, namilumab, and TJ003234) are currently used. Mavrilimumab, a humanized IgG4 mAb, inhibits macrophage polarization by blocking the GM‐CSF receptor. Five clinical studies (NCT04447469, NCT04492514 NCT04463004, NCT0439998, and NCT04397497) have been conducted to evaluate its safety and efficacy in COVID‐19. Two clinical reports showed that mavrilimumab therapy was well tolerated and associated with improved hyperinflammation in COVID‐19 patients, but larger trials are still needed.^376^, ^377^ Gimsilumab acts to block GM‐CSF receptors. One clinical study was conducted without results on its ability to improve systemic inflammation and mortality in COVID‐19 patients.^378^ Otilimab is a human mAb commonly used in RA that blocks the binding of GM‐CSF to the receptor. Currently, otilimab completed one clinical trial (NCT04376684). Lenzilumab, namilumab, and TJ003234 are neutralizing antibodies to GM‐CSF. Lenzilumab has been approved by the FDA for emergency single‐use in COVID‐19 patients, and a phase III study is underway (NCT04351152). One clinical study was conducted with TJ003234 (NCT04341116), and namilumab is not yet available.

#### 4.4.6 TGF‐β intervention therapy

We already know that TGF‐β is involved in the pathogenesis of COVID‐19 and is a key factor released during viral infection and inflammation. Targeting TGF‐β could be a new interventional therapy. Early reports have suggested using antiactive TGF‐β antibodies or TGF‐β inhibitors to treat COVID‐19.^202^ Pirfenidone is a common drug with antifibrotic, anti‐inflammatory, and antioxidant effects.^379^ It acts to downregulate inflammatory cytokine levels and TGF‐β gene expression, as well as to inhibit the conversion of hyperglycosylated pro‐TGF‐β to the latent state by blocking furin.^380^, ^381^, ^382^ The peroxisome proliferator‐activated receptors include PPAR‐α, PPARβ/δ, and PPARγ, and act on the inhibition of inflammatory pathways.^383^, ^384^ PPAR agonists have also been suggested as candidates for the treatment of COVID‐19.^283^, ^385^ Pirfenidone can activate PPARs to exert protective effects in COVID‐19.^386^ Thus, pirfenidone treats COVID‐19 by blocking the production of TGF‐β and activating the protective effect of PPARs, and it is also suggested to be used in combination with NAC. RGD‐binding integrins are major regulators of TGF‐β activation, and it has also been proposed that the RGD motif in the Spike protein of SARS‐CoV‐2 can promote infection through RGD‐binding integrins.^387^, ^388^ A review detailed the role of RGD‐binding integrins on TGF‐β and summarized the potential effects of eight RGD‐binding elements on COVID‐19.^389^ The authors list a number of drugs that clinically target RGD‐binding integrins or TGF‐β, either directly or indirectly, to inform the treatment of COVID‐19. A recent study reported that GLPG‐0187, an integrin/TGF‐β1 inhibitor, blocks SARS‐CoV‐2 Delta and Omicron pseudovirus infection of airway epithelial cells, effectively alleviating the severity of COVID‐19.^390^ In addition, bone morphogenetic protein (BMP) signaling is the opposite pathway of TGF‐β. It has been proposed to counteract the deleterious effects of TGF‐β by activating the BMP pathway, which provides a valuable reference for the treatment of multiorgan damage in COVID‐19.^391^

#### 4.4.7 VEGF inhibitors

The mechanism of action of anti‐VEGF drugs mainly includes direct inhibition of VEGF, influence on VEGF through inhibition of IL‐6, and indirect inhibition of VEGF. Bevacizumab is a humanized anti‐VEGF mAb that is widely used to treat a variety of cancers.^392^

The use of bevacizumab to treat COVID‐19‐mediated inflammation and ARDS is ongoing, and five clinical studies have been registered (NCT04305106, NCT04344782, NCT04954014, NCT04275414, and NCT04822818). The results of one of these clinical studies have been published (NCT04275414). The results show that bevacizumab is effective in improving oxygenation and oxygen support status and has clinical benefit in the treatment of severe COVID‐19 patients.^393^ IL‐6 is an inducer of VEGF production and the use of IL‐6 inhibitors (e.g., siltuximab, see Section 4.4.1 for details) may indirectly reduce VEGF.^394^ Cyclosporine is an immunosuppressant that modulates cAMP signaling and downregulates T‐cell activity and VEGF, cytokine production by inhibiting calcium‐regulated neurophosphatase.^395^ Some studies have reported that low doses of cyclosporine may have therapeutic benefit for COVID‐19, but more data are needed to support this.^396^ There are currently six major clinical trials evaluating the efficacy and safety of cyclosporine in COVID‐19 (NCT04492891, NCT04979884, NCT04451239, NCT04492891, NCT04979884, and NCT04451239).

## 5 CONCLUSION AND DISCUSSION

COVID‐19 is an ongoing pandemic that has created a global health emergency and provided unprecedented challenges for prevention and therapy. Normally, activation of the immune system is one of the important mechanisms of the body's defense against invading pathogens, but uncontrolled immune responses lead to excessive production of inflammation and CS. Multiple disruptive signaling pathways have been implicated in COVID‐19 pathogenesis. Excessive activation of multiple inflammatory pathways by SARS‐CoV‐2 leads to multiorgan damage, CS, and ultimately death. In addition, the high mutability of SARS‐CoV‐2 and the continuous evolution of new variants pose significant challenges for the detection, prevention, and treatment of COVID‐19. The latest Omicron variants are more infectious and induce greater immune escape in humans.^397^ And Omicron circumvents many mAb drugs, such as imdevimab, casirivimab, bamlanivimab, and etesevimab, and reduces their therapeutic activity. WHO approved vaccines using COVID‐19, such as: mRNA‐1273 (Moderna), BNT16b2 (Pfizer BioNTech), AZD1222 (AstraZeneca), Sinopharm (Beijing), and Sinovac (CoronaVac) against Omicron with humoral immunity and neutralizing titers significantly reduced, which undoubtedly poses a test for the future of COVID‐19 control. Currently, the main preventive strategies against the Omicron variant are to strengthen public health precautions for timely detection, early detection, and isolation; to enhance immune resistance by developing new vaccination strategies as the original vaccine remains effective against Omicron; and to use or design new monoclonal antibodies, such as the approved Sotrovimab, Paxlovid, Veklury (raltegravir), molnupiravir, and bebtelovimab for the treatment of moderate or severe COVID‐19 patients.^397^ This paper suggests that the immunopathogenesis of both SARS‐CoV‐2 and its latest variants is through the induction of a dysregulated inflammatory response that promotes CS, ARDS, and multiorgan damage, and therefore, targeting the initiation of inflammatory signaling pathways and the release of downstream cytokines has important benefits for controlling the disease process and adjuvant therapy.

Therefore, therapeutic targets of precise inflammatory pathways are of high value for COVID‐19. There is growing evidence that viral infection disrupts host immune sensing pathways (TLR and NLRP3), disrupts inflammatory signaling pathways (JAK/STAT, NF‐κB, MAPK, P13/Akt/mTOR, and BTK), induces proinflammatory cytokines (IL‐1, IL‐6, TNF‐α, and GM‐CSF), and excessive growth factor release (TGF‐β and VEGF). Therefore, this paper reviews the key inflammatory pathways and targets in the pathogenesis of COVID‐19 and summarizes the targeted intervention therapies in detail. We also believe that drugs targeting inflammatory pathways and their downstream signals and factors, including JAK inhibitors, TLR modulators, and IL‐6, IL‐1 cytokine monoclonal antibodies, may play a more effective role in the prevention and treatment of COVID‐19 in combination with new crown vaccines or antiviral drugs (corticosteroids and raltegravir). Though many drug candidates have shown promising results in the clinic, more in‐depth studies are still needed to decipher the SARS‐CoV‐2‐induced inflammatory pathways and related therapeutic options.

## CONFLICT OF INTERESTS

The authors declare that there are no potential competing interests.

## AUTHOR CONTRIBUTIONS

Yujie Jiang conceived, wrote, and edited the manuscript. Tingmei Zhao and Xueyan Zhou reviewed and revised the important intellectual content. Yu Xiang collected the literature. Xuelei Ma provided guidance on the article outline, the division of content and the details of submission. Gutierrez‐Castrellon gave guidance on the English writing.

## ETHICS APPROVAL STATEMENT

The authors declare that ethics approval was not needed for this study.

6

## Data Availability

Not applicable.
